# Mapping connections in signaling networks with ambiguous modularity

**DOI:** 10.1038/s41540-019-0096-1

**Published:** 2019-05-23

**Authors:** Daniel Lill, Oleksii S. Rukhlenko, Anthony James Mc Elwee, Eugene Kashdan, Jens Timmer, Boris N. Kholodenko

**Affiliations:** 1 Institute of Physics, University of Freiburg, Freiburg, Germany; 2 Systems Biology Ireland, University College Dublin, Dublin, Ireland; 3 School of Mathematics and Statistics, University College Dublin, Dublin, Ireland; 4 BIOSS Centre for Biological Signaling Studies, University of Freiburg, Freiburg, Germany; 5 Conway Institute of Biomolecular & Biomedical Research, University College Dublin, Dublin, Ireland; 6 School of Medicine and Medical Science, University College Dublin, Dublin, Ireland; 7 Department of Pharmacology, Yale University School of Medicine, New Haven, CT USA

**Keywords:** Biochemical networks, Applied mathematics, Computer modelling

## Abstract

Modular Response Analysis (MRA) is a suite of methods that under certain assumptions permits the precise reconstruction of both the directions and strengths of connections between network modules from network responses to perturbations. Standard MRA assumes that modules are insulated, thereby neglecting the existence of inter-modular protein complexes. Such complexes sequester proteins from different modules and propagate perturbations to the protein abundance of a downstream module retroactively to an upstream module. MRA-based network reconstruction detects retroactive, sequestration-induced connections when an enzyme from one module is substantially sequestered by its substrate that belongs to a different module. Moreover, inferred networks may surprisingly depend on the choice of protein abundances that are experimentally perturbed, and also some inferred connections might be false. Here, we extend MRA by introducing a combined computational and experimental approach, which allows for a computational restoration of modular insulation, unmistakable network reconstruction and discrimination between solely regulatory and sequestration-induced connections for a range of signaling pathways. Although not universal, our approach extends MRA methods to signaling networks with retroactive interactions between modules arising from enzyme sequestration effects.

## Headline: Reconstruction of signaling networks with ambiguous modularity

One of the great challenges in biology is to reconstruct the structure of signaling networks from experimental data. Modular Response Analysis (MRA) is a precise method to reconstruct and quantify activating or inhibitory strengths of the connections between network modules called nodes. Standard MRA assumes that each node is unique and no components are shared between different nodes. However, some nodes can include protein complexes with individual proteins from different nodes, for instance a kinase that phosphorylates a substrate which then becomes part of another node. This physical mass transfer can lead to the inference of wrong connections. Here, we extend MRA to account for such situations by discriminating between regulatory connections and connections due to mass transfer. This improvement allows for unmistakable network reconstruction for a range of signaling pathways, and paves the way to a deeper understanding of network structures and their dynamic changes during signal processing.

## Introduction

The reconstruction of connections in signaling networks from experimental data is a key problem in systems biology. An intrinsic challenge in capturing direct network connections is that a signal originating from a component first causes changes in its immediate targets, but then rapidly propagates through the entire network, producing widespread (global) changes that mask direct (local) connections between nodes. Many groups around the world have suggested approaches to reconstruct direct, causative connections between molecules.^1–6^ For signaling and gene networks, Modular Response Analysis (MRA) was developed to infer immediate connections (termed local responses) from the steady state global responses to perturbations.^7–11^ To reduce the vast complexity of signaling networks, MRA divides these networks into modules connected through so-called communicating species, which affect the species dynamics in other modules.^7,12^ Therefore, in a modular framework each network node can be a single species or a module containing internal species interacting within this module. To infer connections between modules, each module is perturbed either alone or together with other modules, and the steady state responses of all communicating species are measured.

MRA neglects mass transfer between network modules, assuming that signaling involves only information transfer.^7,13^ However, activation or inhibition of signaling proteins commonly occurs through posttranslational modifications (PTMs) carried out by enzymatic reactions, such as reactions catalyzed by kinases and phosphatases. These reactions can create mass transfer between modules, if a communicating species (e.g., a kinase) from one module binds to a species from another module, forming a protein–protein complex. When a considerable fraction of a communicating protein is sequestered in a complex that contains species from two different modules, this complex cannot be neglected. The word retroactivity was coined to describe this effect.^14–17^ This has also been referred to as protein sequestration, for instance, the sequestration of an active kinase by its substrate.^18^ Interactions between modules that solely occur as a result of protein sequestration differ from regulatory interactions that activate or inhibit proteins through PTMs. We will term the former as sequestration-induced connections.

When protein moiety conservation includes species from two modules, a perturbation to only one of the species that form a complex bridging two modules will affect both modules. As a result, the modular structure is lost, leading to a breakdown of inter-modular insulation, which is required by MRA. In this case, network reconstruction becomes an ill-posed problem,^19^ and the inferred network topology might depend on particular perturbations that are used for its reconstruction as was recently shown.^20^ Because MRA and its statistical derivatives (e.g. the maximum likelihood and Bayesian MRA) have become broadly applied network reconstruction methods,^4,8–11,21–32^ it is necessary to find out if causative regulatory connections can be precisely inferred in case of extensive retroactivity interactions between modules.

Here we explore how inter-modular protein sequestration affects MRA-based network inference. We show that additional measurements of perturbation-induced changes in inter-modular protein complexes can be exploited to accurately infer network circuitries. An approach is proposed that restores modular insulation by defining communicating species as weighted sums of free species and inter-modular complexes. Similarly to the original work where MRA was developed,^7^ we use mathematical models of signaling pathways to simulate network responses to perturbations, but our approach is solely based on experimental data of responses to perturbations (such as changes in the conserved protein abundances) and it is model-independent. When applicable, this approach allows us to discriminate between solely regulatory network connections (e.g., mediated by protein phosphorylation) and enzyme sequestration-induced connections. We analyze limitations of the proposed MRA extension and cases when it can be efficiently applied.

## Results

### MRA requires the condition of insulation for different modules

A signaling network can often be described by ordinary differential equations,

1 $$\frac{{{\mathrm{d}}z_l}}{{{\mathrm{d}}t}} = \dot z_i = g_l\left( {z_1, \ldots ,z_L,{\boldsymbol{p}}} \right),\quad l = 1, \ldots ,L$$

where *z*_*l*_ are the concentrations of components, such as genes or different protein forms, the function *g*_*l*_ includes the *z*_*l*_ production and consumption rates, and ***p*** is a vector of parameters, such as stoichiometric coefficients and rate constants. It is assumed that only linearly independent concentrations are considered in Eq. (1), and, therefore, the Jacobian matrix has full rank *L*. Consequently, the parameter vector ***p*** can also contain the total abundances of different protein forms that are constrained by moiety conserved cycles.^12^ We consider steady-state conditions and steady-state responses to parameter ***p*** perturbations.

MRA conceptually partitions the network into *N* *≤* *L* modules. A module contains a group of genes or signaling components, which together perform one or more identifiable tasks.^7^ Each module *i* can harbor *m*_*i*_ (*m*_*i*_ ≥ 0) internal species (*y*_*ik*_) and contains a communicating species (*x*_*i*_), which represents the module output. At a steady state $(\dot y_{ik} = 0)$, internal species of each module (*i*) can be expressed as functions (*h*_*ik*_) of the communicating species and parameters,

2 $$y_{ik} = h_{ik}\left( {x_1, \ldots ,x_N,{\boldsymbol{p}}} \right)\quad i = 1, \ldots ,N\quad k = 1, \ldots ,m_i$$

Equation (2) allows us to use a smaller set of *N* algebraic equations, which governs the steady state behavior of module outputs (*x*_*i*_), which become nodes of a modular network,

3 $$g_i\left( {h_{11}, \ldots ,h_{Nm_N},x_1, \ldots ,x_N,{\boldsymbol{p}}} \right) = f_i\left( {x_1, \ldots ,x_N,{\boldsymbol{p}}} \right) = 0\quad i = 1, \ldots ,N$$

We quantify a direct connection from module *j* to module *i* by a relative change (Δ*x*_*i*_/*x*_*i*_) in the activity of communicating species *x*_*i*_ of module *i* brought about by a change (Δ*x*_*j*_/*x*_*j*_) in the output activity *x*_*j*_ of module *j*, provided that these two modules are conceptually isolated from the network. This condition implies that all other modules except these two remain unperturbed (∂*x*_*k*_ = 0, *k* ≠ *i*, *j*), whereas the affected module *i* is allowed to relax to its steady state.^7,13^ Under this condition, the ratio $r_{ij} = \partial {\mathrm{ln}}\,x_i/\partial {\mathrm{ln}}\,x_j$ can be found via implicit differentiation of the function *f*_*i*_ in Eq. (3).

4 $$\begin{array}{*{20}{l}} {\partial f_i} ={\mathop {\sum}\limits_k {\frac{{\partial f_i}}{{\partial x_k}}} \partial x_k = \frac{{\partial f_i}}{{\partial x_i}}\partial x_i + \frac{{\partial f_i}}{{\partial x_j}}\partial x_j = 0} \hfill \\ {\partial \ln x_i}={\frac{{\partial x_i}}{{x_i}}\quad \partial {\mathrm{ln}}x_j = \frac{{\partial x_j}}{{x_j}}} \hfill \\ {r_{ij}}={\frac{{\partial {\mathrm{ln}}x_i}}{{\partial {\mathrm{ln}}x_j}} = \frac{{x_j}}{{x_i}}\frac{{\partial x_i}}{{\partial x_j}} = - \frac{{x_j}}{{x_i}}\frac{{\frac{{\partial f_i}}{{\partial x_j}}}}{{\frac{{\partial f_i}}{{\partial x_i}}}},\quad i,j = 1, \ldots ,N} \hfill \end{array}$$

The coefficients *r*_*ij*_ are called the connection coefficients or the local responses and form the connection matrix that determines the direction and strengths of direct network connections.^7,9^ These connection coefficients cannot be immediately measured, because a perturbation to a single module propagates through the network, and the experimentally observed changes in other modules might be indirect.

MRA calculates connection coefficients (*r*_*ij*_) from steady-state responses of an entire network to parameter (*p*_*j*_) perturbations. Experimentally, perturbations use siRNA (affecting protein abundances), inhibitors, drugs and genetic alterations.^23–25,29,30,33^ Resulting steady-state responses are termed the global response coefficients (*R*_*ij*_),^7,13^

5 $$R_{ij} = \left. {\frac{{\partial {\mathrm{ln}}x_i}}{{\partial p_j}}} \right|_{{\mathrm{steady}}\,{\mathrm{state}}},\quad i = 1, \ldots ,N,\quad j = 1, \ldots ,M$$

MRA has developed an experimental design that determines network connections (*r*_*ij*_) by measuring global responses (*R*_*ij*_).^8,9^ A specific feature of this design is selecting a set of experimental interventions that *do not directly* influence the output *x*_*i*_ of module *i* in order to find network connections (*r*_*ij*_) leading to this module *i*. Each of these perturbations may directly affect one or *many* nodes *x*_*k*_ different from *x*_*i*_. Formally, for each *x*_*i*_ (*i* = 1, …, *N*), we choose and then perturb ***N*** − **1** parameters *p*_*j*_ known to have the property that the function *f*_*i*_ in Eq. (3) does not depend upon *p*_*j*_,

6 $$\frac{{\partial f_i\left( {x_1, \ldots ,x_N,{\boldsymbol{p}}} \right)}}{{\partial p_j}} = 0,\quad j = 1, \ldots ,N - 1$$

These *N* − 1 parameters selected for perturbation will be termed perturbation parameters. The condition (Eq. 6) that parameter *p*_*j*_ does not directly affect module *i*, whereas *p*_*j*_ can affect other modules *j* (*j* ≠ *i*) is called the module insulation condition. Usually biological information to select such a parameter *p*_*j*_ is available, for instance, it can be known that an inhibitor of a membrane kinase has no direct influence on a cytoplasmic phosphatase, or the abundance of a certain protein has no direct influence on unrelated biochemical interactions in a different module. Differentiating the function *f*_*i*_ in Eq. (3) with respect to *p*_*j*_ and using the module insulation condition (6) and Eqs. (4) and (5), we arrive at MRA equations (Eq. 7),

7 $$\mathop {\sum}\limits_{k = 1}^{N} {r_{ik}} R_{kj} = 0,\,r_{ii} = - 1,\quad i = 1, \ldots ,N$$

For every module *i*, Eq. (7) determines the connection coefficients ***r***_***ij***_ using the global network responses (***R***_***kj***_) of each module (***k*** = 1, …, ***N***) to perturbations of ***N*** − **1** parameters ***p***_***j***_ (statistical MRA formulations can use less or more than ***N*** − **1** perturbations^4,23–25^). Each of the selected perturbations (parameters ***p***_***j***_ in Eq. 6) cannot directly influence module ***i***, but together these ***N*** − **1** independent perturbations should affect all the other (***N*** − **1**) modules of the network except module ***i***.^7,9^ Importantly, the connection coefficients determined by Eq. (7) do not depend on a particular choice of ***N*** − **1** parameters ***p***_***j***_, provided that the module insulation condition (6) is satisfied for each parameter ***p***_***j***_, ***j*** = 1, …, ***N*** − **1**.^7^ Indeed, connection coefficients are uniquely determined by a system steady state that does not depend on the choice of perturbation parameters, see Eq. (4).

### Violation of insulation condition by complexes of proteins that belong to different modules

Module outputs are often represented by signaling enzymes, such as kinases.^4,23,25^ Suppose a communicating species of module *i*, e.g., a kinase, forms a complex with its substrate that belongs to another module *j*. If the concentration of this complex is comparable with the free concentration of the kinase or its substrate, the complex concentration cannot be neglected. Because protein synthesis and degradation usually occur at much longer timescale than (de)phosphorylation reactions, the total concentrations of different protein forms are conserved and, thus, the protein abundances are parameters of the system. Consequently, at a network steady state, the concentration of a complex containing proteins from two different modules (*i* and *j*) will depend on the total abundances of both these proteins, which will be parameters denoted as, *p*_*i*_ and *p*_*j*_. If we assign the kinase-substrate complex to module *i* that includes the kinase as a communicating species, then a perturbation to parameter *p*_*j*_ (the total concentration of the substrate) will affect not only module *j* but also the free kinase and the complex concentrations, i.e., module *i* (see Supplementary material section 1). Alternatively, if we assign the complex to module *j* that includes the kinase substrate, then a perturbation to parameter *p*_*i*_ (the total kinase concentration) will affect not only module *i* but also the free substrate and the complex concentrations, i.e., module *j*. Consequently, the choice of perturbation parameters as the total protein abundances will violate the module insulation condition (Eq. 6) for one or both of these modules. At the same time, perturbations of the other parameters, such as rate constants of enzymatic reactions, might not violate the module insulation condition.

Sequestration of a kinase (or a phosphatase) from module *i* by a substrate from module *j* means that module *j* retroactively affects module *i*, although module *j* is only a recipient of a signal from module *i*. Proper parameter perturbations that are consistent with Eq. (6) can reveal both regulatory influences and sequestration-induced feedbacks. However, the violation of the module insulation condition might lead to contradictory results of inferring different network circuitries by using different perturbations, as illustrated below and in Section 1 of Supplementary material, using a simple example.

### Using MRA to map network connections when protein complexes bridge modules

**Fig. 1:**
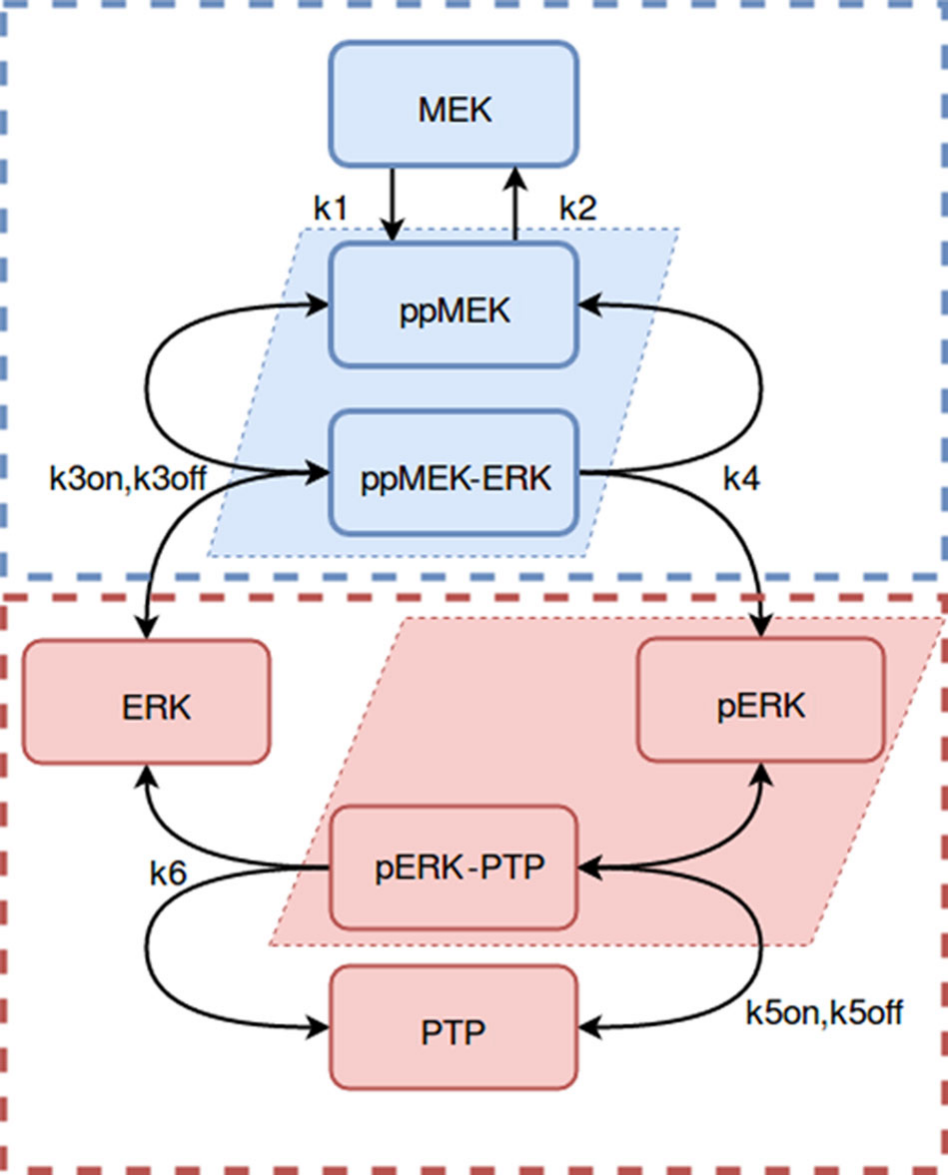
Reaction scheme of the MEK/ERK cascade model studied by Parabakaran et al.^20^ Reaction rates are described by mass action kinetics, the appropriate rate constants are indicated at the arrows. Species of the MEK module are indicated in blue, species of the ERK module are indicated in red. Communicating species are selected as the sums of species in shaded parallelograms (Eq. 9)^20^

We first illustrate the challenges arising from the protein sequestration using paradoxical, at first glance, results of finding distinct network circuitries while perturbing different parameter sets. Prabakaran and colleagues^20^ showed network inference challenges both experimentally, using an in vitro reconstituted system of purified recombinant kinases (RAF, MEK, and ERK) and phosphatases (the serine/threonine phosphatase PP2A and the tyrosine specific phosphatase PTP), and also theoretically using a simplified model of the MEK/ERK cascade (Fig. 1), described as follows. A constant external signal (mimicked by a mutated constitutively active RAF kinase) phosphorylated MEK on two serines in the activation loop, yielding active ppMEK (MEK phosphorylation was considered as one step in the model^20^). MEK phosphatase PP2A was not explicitly considered in the model, and ppMEK dephosphorylation was described by a first order process. Active ppMEK phosphorylated ERK on the tyrosine in the activation loop. The other activating site on ERK, threonine, was mutated to a non-phosphorylatable residue, thus rendering only phosphorylated ERK (pERK) susceptible to dephosphorylation by PTP, yielding ERK. The abundances of MEK, ERK, and PTP were considered constant.^20^

Accordingly, out of 7 network species, only four of the species concentrations were linearly independent (Fig. 1). We can select these species as [*ppMEK*], [*pERK*], $\left[ {ppMEK \cdot \cdot ERK} \right]$ and $\left[ {pERK \cdot \cdot PTP} \right]$ (indicated by the shaded quadrilaterals in Fig. 1), whose dynamics is governed by Eq. (8). The remaining linearly dependent concentrations, [*MEK*], [*ERK*], and [*PTP*], are expressed through moiety-conservation laws using the total protein abundances, *ERK*^*tot*^, *MEK*^*tot*^, and *PTP*^*tot*^,

8 $$\begin{array}{l}d\left[ {ppMEK} \right]/dt = k_1 \cdot \left[ {MEK} \right] - k_2 \cdot \left[ {ppMEK} \right] - k_3^{on} \cdot \left[ {ppMEK} \right] \cdot \left[ {ERK} \right]\\ + \,k_3^{off} \cdot \left[ {ppMEK \cdot \cdot ERK} \right] + k_4 \cdot \left[ {ppMEK \cdot \cdot ERK} \right]\\ d\left[ {ppMEK \cdot \cdot ERK} \right]/dt = k_3^{on} \cdot \left[ {ppMEK} \right] \cdot \left[ {ERK} \right]\\ - \,k_3^{off} \cdot \left[ {ppMEK \cdot \cdot ERK} \right] - k_4 \cdot \left[ {ppMEK \cdot \cdot ERK} \right]\\ d\left[ {pERK} \right]/dt = k_4 \cdot \left[ {ppMEK \cdot \cdot ERK} \right]\\ - \,k_5^{on} \cdot \left[ {pERK} \right] \cdot \left[ {PTP} \right] + \,k_5^{off} \cdot \left[ {pERK \cdot \cdot PTP} \right]\\ d\left[ {pERK \cdot \cdot PTP} \right]/dt = k_5^{on} \cdot \left[ {pERK} \right] \cdot \left[ {PTP} \right]\\ - k_5^{off} \cdot \left[ {pERK \cdot \cdot PTP} \right] - k_6 \cdot \left[ {pERK \cdot \cdot PTP} \right]\\ \left[ {MEK} \right] = MEK^{tot} - \left[ {ppMEK} \right] - [ppMEK \cdot \cdot ERK]\\ \left[ {ERK} \right] = ERK^{tot} - \left[ {ppMEK \cdot \cdot ERK} \right] - \left[ {pERK} \right] - [pERK \cdot \cdot PTP]\\ \left[ {PTP} \right] = PTP^{tot} - [pERK \cdot \cdot PTP]\end{array}$$

This model MEK/ERK cascade was divided into two modules (highlighted in pink and blue colors in Fig. 1). The total concentration of phosphorylated MEK and ERK (including protein–protein complexes) were chosen as module outputs - communicating species *x*_1_ and *x*_2_,^20^

9 $$\begin{array}{l}x_1 = [ppMEK] + [ppMEK \cdot \cdot ERK]\\ x_2 = [pERK] + [pERK \cdot \cdot PTP]\end{array}$$

To infer network interactions, the MEK module was perturbed by varying the total MEK abundance (*MEK*^*tot*^), and the ERK module was perturbed by varying either the total ERK abundance (*ERK*^*tot*^) or the total PTP abundance (*PTP*^*tot*^). Surprisingly, the circuitries of the reconstructed networks were found different for these two different sets of perturbations.^20^ Perturbation of *ERK*^*tot*^ revealed an activating influence of ERK on MEK (manifested by positive connection coefficient *r*_12_), while perturbation of *PTP*^*tot*^ revealed an inhibiting influence of ERK on MEK (negative connection coefficient *r*_12_).^20^

Whereas the inhibiting influence of *ERK* on *MEK* can be interpreted as the sequestration of active *MEK* by inactive *ERK* within the $ppMEK \cdot \cdot ERK$ complex, the inferred activating influence of *ERK* on *MEK* is clearly a false positive result for this in vitro reconstituted network. Moreover, this MEK/ERK network has only one non-zero regulatory connection, a connection from MEK to ERK (positive connection coefficient *r*_21_). These findings by Prabakaran and colleagues^20^ become less surprising, if we recall that assigning the $ppMEK \cdot \cdot ERK$ complex to either *MEK* module or *ERK* module violates the module insulation condition (6). Next, we demonstrate how the unique circuitry of this network can unequivocally be inferred, using the same perturbations that led to the inconsistent topologies determined by Prabakaran and colleagues.^20^

A system of equations governing steady state behavior of the communicating species *x*_1_ and *x*_2_ (Eq. 9) is derived using Eq. (8). For purposes of readability, only arguments of the governing functions (*f*_1_ and *f*_2_) are presented below. Full expressions for these equations can be found in Supplementary material (section 2.1).

10 $$\begin{array}{l}f_1 = f_1\left( {x_1,x_2,MEK^{tot},ERK^{tot},k_1,k_2,k_3^{on},k_3^{off},k_4} \right) = 0\\ f_2 = f_2\left( {x_1,x_2,ERK^{tot},PTP^{tot},k_3^{on},k_3^{off},k_4,k_5^{on},k_5^{off},k_6} \right) = 0\end{array}$$

Equation (10) shows that both functions *f*_1_ and *f*_2_ depend on the ERK abundance. Therefore when the communicating species are selected according Eq. (9), a perturbation to *ERK*^*tot*^ not only directly affects the ERK module (*f*_2_) but also immediately perturbs the MEK module, because ∂*f*_1_/∂*ERK*^*tot*^ ≠ 0, violating Eq. (6). Likewise, perturbations to the rate constants of the ppMEK-ERK complex formation/dissociation $(k_3^{on},k_3^{off},k_4)$ also violate Eq. (6), because the governing functions for both modules depend on these parameters. At the same time, perturbations of parameters, which are the rate constants of other reactions (intrinsic to single modules) $(k_1,k_2,k_5^{on},k_5^{off},k_6)$ and the PTP abundance (*PTP*^*tot*^), do not violate the insulation condition (6) for both communicating species (module outputs) *x*_1_ and *x*_2_. Perturbations of *any* two parameters from this set will allow the inference of the unique, true network circuitry with an activating connection from MEK to ERK and sequestration feedback from ERK to MEK (see Supplementary material, section 2).

We can envision the situation when a selected parameter (*p*_*j*_) might directly affect internal species but does not influence the module output, i.e. a communicating species (*x*_*i*_). Using Eqs. (2) and (3), the insulation condition (6) for module *i* can be reformulated in terms of the derivatives of its internal species, *y*_*ik*_ = *h*_*ik*_(*x*_1_, …, *x*_*N*_, ***p***), with respect to *p*_*j*_,

11 $$\frac{\partial f_i\left( {x_1, \ldots ,x_N,{\boldsymbol{p}}} \right)}{{\partial p_j}} = \frac{\partial g_i\left( {h_{11}, \ldots ,h_{Nm_N},x_1, \ldots ,x_N,{\boldsymbol{p}}} \right)}{\partial p_j} = \mathop {\sum}\limits_{m,k} {\frac{\partial g_i}{\partial h_{mk}}} \cdot \frac{\partial h_{mk}}{\partial p_j} + \frac{\partial g_i}{\partial p_j} = 0$$

Formally, Eq. (11) allows the dependence of some internal species (*y*_*ik*_ = *h*_*ik*_) on *p*_*j*_ provided that the sum of the partial derivatives in Eq. (11) equals 0. Thus, within the MRA framework a perturbation to parameter *p*_*j*_ can be applicable for inferring connections (*r*_*ij*_) leading to this module *i* even if internal species of module *i* are directly perturbed, but the governing equation *f*_*i*_ and, thus, the communicating species *x*_*i*_ are not directly perturbed. Clearly, the given choice of a communication intermediate (that determines its governing function) also informs if the selected parameter violates or does not violate the module insulation condition (Eqs. 6 and 11).

Because perturbations to the protein abundances, using siRNA or irreversible covalently-bound inhibitors are commonly used, we might ask whether alternative choices of the communicating species (i.e., the module outputs) can ensure that the module insulation condition holds for both ERK and PTP abundance perturbations. Assuming that the absolute or relative (see Supplementary material, sections 2 and 3) changes in the concentrations of both free ppMEK and the ppMEK-ERK complex can be individually measured, we introduce a new communicating species of the MEK module, $x_1^a$ that depends on a free non-negative parameter *a*, while keeping the same ERK module output, *x*_2_, as follows (cf. Eq. 9),

12 $$\begin{array}{l}x_1^a = \left[ {ppMEK} \right] + a \cdot [ppMEK \cdot \cdot ERK]\\ x_2 = \left[ {pERK} \right] + \left[ {pERK \cdot \cdot PTP} \right]\end{array}$$

This new MEK module output, $x_1^a$, is the weighted sum of the concentrations of the free active enzyme and the enzyme-substrate complex, which is multiplied by a free weight parameter *a*. If *a* = 0, $x_1^a$ is the free form of active MEK. If *a* = 1, $x_1^a$ is the total concentration of the phosphorylated MEK forms, the communicating intermediate selected by Prabakaran and colleagues.^20^ If *a* → ∞, then only the ppMEK-ERK complex acts as a communicating species.

The rationale behind selecting the new module MEK output $x_1^a$ is the following. A perturbation, e.g., an increase in *ERK*^*tot*^ leads to an increase in the free ERK concentration and the ppMEK-ERK complex, but to a decrease in free ppMEK (sequestered by ERK). Because $x_1^a$ is chosen as a linear combination of ppMEK and ppMEK-ERK, at some value of the weight parameter *a*, these opposite changes of the terms within the communicating species $x_1^a$ will cancel each other out. At this *a* = *a*^*opt*^ value, perturbations to the ERK abundance will no longer directly affect the module MEK output $x_1^{a^{opt}}$. The expression for *a*^*opt*^ can be obtained by solving the module insulation condition (Eq. 11), after substituting *p*_*j*_ = *ERK*^*tot*^ and the governing function for $x_1^a$ given by Eq. (12) (see Supplementary material, section 2.1 for derivation),

13 $$a^{opt} = k_1/(k_1 + k_2)$$

Consequently, at *a* = *a*^*opt*^, we obtain the following dependencies of the governing functions of communicating species $x_1^{a^{opt}}$ and *x*_2_ on parameters (see Supplementary material, section 2 for details),

14 $$\begin{array}{l}f_1^{a^{opt}} = f_1^{a^{opt}}\left( {x_1^{a^{opt}},x_2,MEK^{tot},k_1,k_2} \right)\\ f_2^{a^{opt}} = f_2^{a^{opt}}\left( {x_1^{a^{opt}},x_2,ERK^{tot},PTP^{tot},k_1,k_2,k_3^{on},k_3^{off},k_4,k_5^{on},k_5^{off},k_6} \right)\end{array}$$

It follows from Eq. (14), that the insulation condition (Eq. 6) is satisfied for perturbations to any of the three conserved total abundances, *MEK*^*tot*^, *PTP*^*tot*^, *ERK*^*tot*^, resulting in a unique MRA-reconstructed network circuitry. However, perturbations to the rate constants (*k*_1_, *k*_2_) now violate Eq. (6) in contrast to the case of the initially selected communicating species of the MEK module (Eqs. 9 and 10). At the same time, perturbations to the rate constants $(k_5^{on},k_5^{off},k_6)$ internal to the ERK module do not contradict Eq. (6). This is supported by numerical calculations that solve Eq. (7), - the MRA equations, using simulated network perturbation responses, *R*_*ij*_, which normally would be obtained experimentally. The results demonstrate that for the governing functions defined by Eq. (14), perturbations to any parameters, *MEK*^*tot*^, *ERK*^*tot*^, *PTP*^*tot*^, $k_3^{on}$, $k_3^{off}$, *k*_4_, $k_5^{on}$, $k_5^{off}$, and *k*_6_, do not break up the module insulation condition, thus resulting in the reconstruction of the quantitatively identical matrices for the connections coefficients and the unmistakable network circuitry (see Supplementary material, Eqs. 18 and 19). We will term parameter perturbations, which do not violate the insulation condition, permissible perturbations (see Eqs. 6 and 11).

**Fig. 2:**
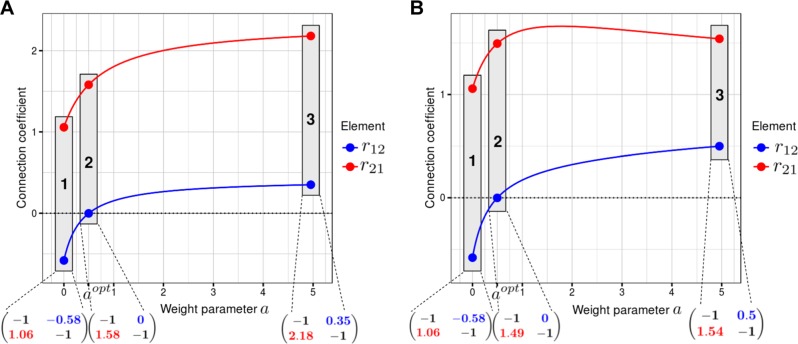
Dependence of elements of the connection matrix *r*_*ij*_ on the weight parameter *a*. The regulatory connection *r*_21_ is depicted in red. The sequestration-induced (aka retroactivity) connection *r*_12_ that changes its sign with the increase in the weight parameter *a* is depicted in blue. MEK and ERK module outputs were defined by **a** Eq. (12) or **b** Eq. (15). In both cases, the total concentrations of MEK and ERK (*MEK*^*tot*^ and *ERK*^*tot*^, respectively) were perturbed. The connection matrices are shown for different weight parameter values, *a* = 0 (point 1), *a* = *a*^*opt*^ (2), and *a* = 5 (point 3). Diagonal elements are always equal to −1^7^

Interestingly, the choice of $x_1^{a^{opt}}$ (when *a* = *a*^*opt*^) results in the connection coefficient *r*_12_ (which determines the sequestration feedback and is calculated by solving Eq. 7) equal to zero. Consequently, by changing the communicating species (module outputs), regulatory connections in this network can be distinguished from solely retroactivity connections induced by protein sequestration. This is illustrated in Fig. 2a, showing how the regulatory activating connection from MEK to ERK (coefficient *r*_21_, the red curve) and sequestration feedback connection from ERK to MEK (coefficient *r*_12_, the black curve) vary with the change in the weight parameter *a*. Using Eq. (7), these connection coefficients (Fig. 2a) are obtained following small perturbations of the protein abundances (*MEK*^*tot*^ and *ERK*^*tot*^) and numerical calculations of perturbation responses for the module outputs that are given by Eq. (12). The only requirement for the corresponding experimental set-up is the separate determination of the ppMEK-ERK complex response and ppMEK response (here these responses are simulated using a model but they would be obtained experimentally for the normal MRA inference procedure^7^). Importantly, the perturbation responses need to be measured experimentally or simulated computationally only once, whereas the connection coefficients are calculated for a range of different values of the weight parameter *a*, using the MRA equations (Eq. 7). At *a* = 0 (point 1 on Fig. 2a), the MEK module communicating species is free active MEK (ppMEK), *r*_21_ is positive because ppMEK activates ERK, whereas *r*_12_ is negative, reflecting the sequestration of ppMEK by ERK. Importantly, with an increase in the weight parameter *a* > 0, the negative sequestration connection coefficient *r*_12_ increases, assumes 0 at *a* = *a*^*opt*^ (point 2 in Fig. 2a) and then changes its sign, further increasing with increasing *a*. In fact, at *a* = 1, *r*_12_ is surprisingly positive, as found for *MEK*^*tot*^ and *ERK*^*tot*^ perturbations by Prabakaran and colleagues^20^ (Fig. 2a). Both coefficients *r*_21_ and *r*_12_ reach positive values when *a* tends to infinity (point 3 in Fig. 2a).

In Eq. (12), only one module (MEK) output was selected as a weighted sum of the free active kinase (ppMEK) and the kinase-substrate complex (ppMEK-ERK). However, in the absence of prior information about which protein in the complex is an enzyme and which is a substrate, we can add the concentration of the ppMEK-ERK complex (scaled by a parameter *a*) to both module outputs,

15 $$\begin{array}{l}x_1^a = \left[ {ppMEK} \right] + a \cdot [ppMEK \cdot \cdot ERK]\\ x_2^a = \left[ {pERK} \right] + \left[ {pERK \cdot \cdot PTP} \right] + a \cdot [ppMEK \cdot \cdot ERK]\end{array}$$

This symmetrical choice of module outputs also allows us to uniquely infer the network circuitry, eliminating retroactivity connection coefficient at the same value of the weight parameter *a* = *a*^*opt*^ given by Eq. (13). Moreover, for the module outputs given by Eq. (15), the governing functions $f_1^{a^{opt}}$ and $f_2^{a^{opt}}$ will depend on the same parameter sets presented in Eq. (14) (although the expressions for these functions will change, see Supplementary material, section 2). Therefore similarly as above, perturbations to any two parameters in the following set, *MEK*^*tot*^, *ERK*^*tot*^, *PTP*^*tot*^, $k_3^{on}$, $k_3^{off}$, *k*_4_, $k_5^{on}$, $k_5^{off}$, and *k*_6_, will reveal a unique network circuitry (see Supplementary material, section 2.1). For the choice of module outputs given in Eq. (15) and perturbations to *MEK*^*tot*^ and *ERK*^*tot*^, Fig. 2b illustrates the dependencies of the connection coefficients on the parameter *a*. However, the asymptotic behavior (at large *a* values) of the connection coefficients is different. Now both *r*_12_ and *r*_21_ approach 1, when *a* → ∞, because the ppMEK-ERK complex becomes a main output for both MEK and ERK modules (*r*_12_ and *r*_21_ equal to 1 merely describes the influence of the ppMEK-ERK complex on itself).

Summarizing, by measuring the changes in the free active kinase (ppMEK) and the kinase-substrate complex (ppMEK-ERK) concentrations separately upon perturbations to any two protein abundances (*MEK*^*tot*^ and *ERK*^*tot*^ or *MEK*^*tot*^ and *PTP*^*tot*^), MRA can precisely reconstruct the signaling network analyzed by Prabakaran and colleagues,^20^ distinguishing between regulatory and retroactive connections and avoiding the inconsistent topologies.

### A model of three-tier cascade with no regulatory feedback connections

**Fig. 3:**
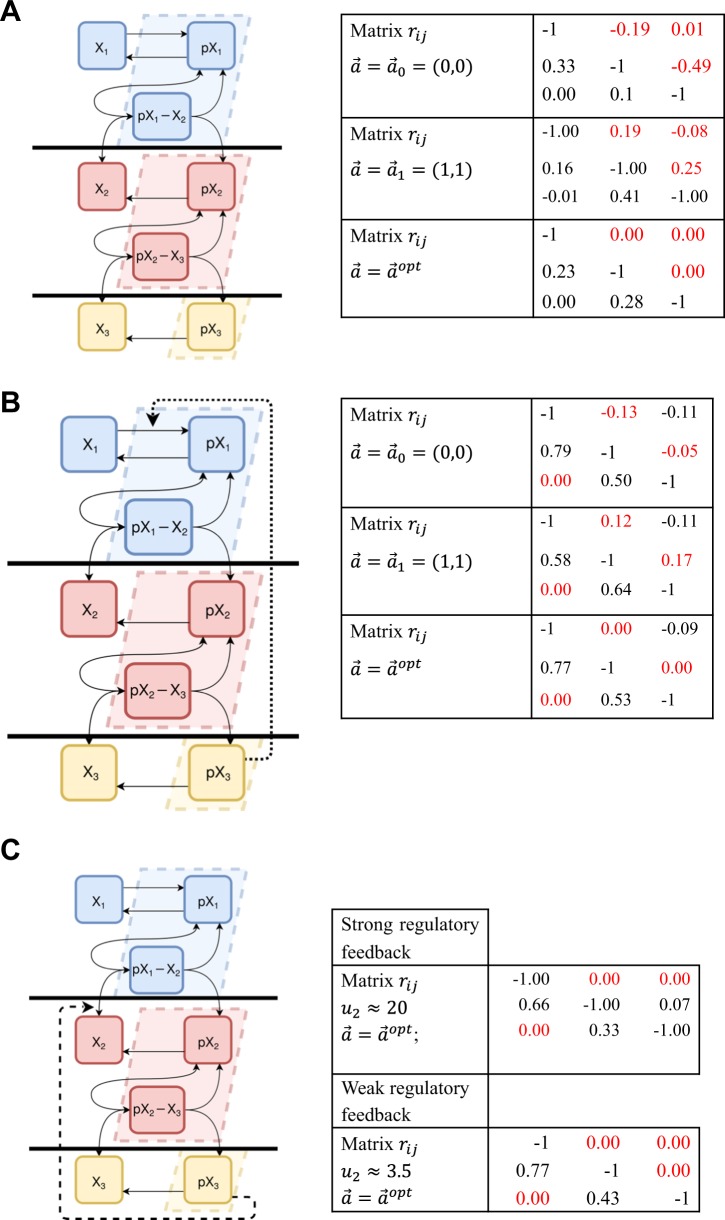
Reconstruction of connection matrices for three-tier cascades. Cascade modules are indicated by different colors and separated by bold horizontal lines for illustrative purposes. Dashed parallelograms indicate substances that are included into module outputs (Eq. 18). For network reconstruction, the total protein abundances, $X_1^{tot}$, $X_2^{tot}$, and $X_3^{tot}$, were perturbed. **a** Left panel: Scheme of a 3-tier cascade without regulatory feedback loops. Right: Reconstructed matrices of connections coefficients (*r*_*ij*_) for different weight parameters *a*_*i*_ (including $a_i^{opt}$). **b** Left: Scheme of a 3-tier cascade with a regulatory feedback from module 3 to module 1. Right: reconstructed connection matrices *r*_*ij*_ for different weights *a*_*i*_. **c** Left: scheme of a 3-tier cascade with a regulatory feedback from module 3 to module 2, which are also connected through feedforward activation of module 3 by module 2, creating a sequestration feedback. Right: reconstructed connection matrices *r*_*ij*_ for different strengths (*u*_2_) of the positive regulatory feedback and optimal weights $a_i^{opt}$. For all right panels, the matrix elements that correspond to retroactive (i.e. sequestration) connections are depicted in red

Next, using several examples of activating cascades where inter-modular protein-protein complexes are formed both upstream and downstream of a cascade tier, we show that regulatory connections can be unmistakably reconstructed while sequestration connections can be nullified using our method. First, we considered a cascade without regulatory feedback connections (Fig. 3a). As above, we used a mass action kinetic model to simulate steady-state responses of the cascade to perturbations. The kinetic equations of the model and parameter values are presented in Supplementary material (Tables 2 and 3).

The waterfall structure (Fig. 3a) of kinase activation cascades is common for many signal transduction cascades, such as the ERK, p38, JNK, and PI3K/AKT cascades.^34^ Proteins in each tier can be in inactive ([*X*_1_], [*X*_2_], and [*X*_3_]) or active ([*pX*_1_], [*pX*_2_], and [*pX*_3_]) states. The enzyme forms, *pX*_1_ and *pX*_2_, activate inactive enzyme forms, *X*_2_ and *X*_3_, respectively, in a two-step process which involves the formation of an enzyme-substrate complex followed by phosphorylation. The total protein abundances, $X_1^{tot}$, $X_2^{tot}$ and $X_3^{tot}$, are conserved. We divided the cascade into three modules along its tiers, as shown by different colors in Fig. 3a. As above, we considered module outputs, in which the enzyme-substrate complex that bridges two modules was added to a single module (Eq. 18) or to both neighboring modules (Eq. 19),

18 $$\begin{array}{l}x_1^a = \left[ {pX_1} \right] + a_1 \cdot \left[ {pX_1 \cdot \cdot X_2} \right]\\ x_2^a = \left[ {pX_2} \right] + a_2 \cdot \left[ {pX_2 \cdot \cdot X_3} \right]\\ x_3^a = \left[ {pX_3} \right]\end{array}$$

19 $$\begin{array}{l}x_1^{a \ast } = \left[ {pX_1} \right] + a_1 \cdot \left[ {pX_1 \cdot \cdot X_2} \right]\\ x_2^{a \ast } = \left[ {pX_2} \right] + a_1 \cdot \left[ {pX_1 \cdot \cdot X_2} \right] + a_2 \cdot \left[ {pX_2 \cdot \cdot X_3} \right]\\ x_3^{a \ast } = [pX_3] + a_2 \cdot \left[ {pX_2 \cdot \cdot X_3} \right]\end{array}$$

Similarly to the model presented above (Eq. 8), if the concentrations of enzyme-substrate complexes cannot be neglected, the choice of the parameters to be perturbed can affect the inferred network circuitry. A standard selection of model outputs corresponds to weight parameters *a*_*i*_ equal to zero, if only free phosphorylated enzyme forms are considered, or to 1, if the total phosphorylated forms are measured and analyzed. Using this standard selection for all three networks and making perturbations to each of the total protein abundances, we calculated matrices of connection coefficients, *r*_*ij*_, which showed non-zero feedback connections from each downstream module to its upstream module (see two reconstructed connection matrices for weight parameters *a*_*i*_ = 0 and *a*_*i*_ = 1 and the module outputs in Eq. (18) in the right panel of Fig. 3a). However, we do not know whether these connections correspond to regulatory or solely protein sequestration-induced feedbacks. Moreover, reconstructed network circuitries might differ for differently selected sets of perturbation parameters.

To elucidate the nature of inferred connections, we calculated the dependences of the connection coefficients on positive values of weight parameters *a*_*i*_ for two sets of module outputs (Eqs. 18 and 19). We found that the connection coefficients, *r*_12_, *r*_23_ and *r*_13_, changed their signs with the *a*_*i*_ changes, that suggested sequestration connection, whereas connections *r*_21_ and *r*_32_ did not change the sign, indicating regulatory feedforward activation connections. For both choices of module outputs, the same $\vec a = \vec a^{opt}$ values yielded zero values for suggested sequestration connections coefficients, *r*_12_, *r*_23_, and *r*_13_ (see Supplementary material, Eqs. 23 and 24). Also, for both choices of communicating species (Eqs. 18 and 19) we found that at $\vec a = \vec a^{opt}$, the network was uniquely reconstructed using perturbations to wide range of perturbation parameters that included all three total protein abundances ($X_1^{tot}$, $X_2^{tot}$, and $X_3^{tot}$) and numerous kinetic constants (see Supplementary material, section 4). Recapping, at $\vec a = \vec a^{opt}$ the connections coefficients that have changed their sign with changing $\vec a$ become zero, and the connection matrix *r*_*ij*_ becomes invariant to a wide range of applied perturbations.

The results shown in Fig. 3 were obtained numerically, because the equations that govern the steady state behavior of communicating species do not allow for an analytical solution in this case. However, as in the previous example, in which the governing equations (Eq. 14) were solved analytically and numerically, our calculations suggested that nonzero connections, *r*_12_, *r*_23_ and *r*_13_, inferred for the standard selection of model outputs, were solely induced by protein sequestration (retroactivity). Importantly, at the $\vec a^{opt}$ values of the weights *a*_*i*_, the invariance of the connection matrix *r*_*ij*_ with respect to different perturbations was brought about by a restoration of the modular insulation condition (Eq. 6).

In summary, these result suggest that if the connection coefficients *r*_*ij*_ have different signs for different values of weight parameters *a*_*i*_, and these *r*_*ij*_ are nullified at certain values, $a_i^{opt}$, then these $a_i^{opt}$ values restore the modular insulation condition (Eq. 6) for a wide range of perturbations. Consequently, the resulting connection matrix (***r***) becomes invariant with respect to the choice of permissible perturbations. The exact set of permissible perturbations at $\vec a = \vec a^{opt}$, however, may differ, depending on the choice of communicating species.

### A model of three-tier cascade with positive and negative regulatory feedback connections

Signaling cascades considered above did not have regulatory feedback loops. To explore how the regulatory feedbacks can be distinguished from retroactivity, i.e., solely sequestration induced feedbacks, we next analyzed models of three-tier enzymatic cascades with both types of feedback connections. First, we considered cascades with regulatory feedback loops connecting modules, which are not linked by immediate feedforward connections within a waterfall cascade structure. A reaction scheme in Fig. 3b presents a signaling cascade with tiers 1 and 2 that activate their immediate downstream tiers 2 and 3, respectively, and regulatory feedback from tier 3 to tier 1, which can be negative or positive.

For simplicity, we first used a standard Michaelis-Menten description of the regulatory feedback.^35^ Assuming non-competitive activation or inhibition of the reaction of *X*_1_ phosphorylation by an active form (*pX*_3_) of tier 3 enzyme, the reaction rate was multiplied by the following multiplier, (1 + *u*_1_[*pX*_3_]/*k*_9_)/(1 + [*pX*_3_]/*k*_9_).^36^ We can readily see that regulatory feedback from module 3 to module 1 is positive, if *u*_1_ > 1, and it is negative, if 0 < *u*_1_ < 1.

We defined the communicating species using Eq. (18), in which an enzyme-substrate complex that bridges two modules is added to a single module output, or using Eq. (19) where these complexes are added to both neighboring modules. Similarly as above, for either selection of communicating species we found that the connection coefficients, *r*_12_ and *r*_23_, changed their sign at certain values $a_i = a_i^{opt}$, while the connection coefficients, *r*_21_, *r*_32_, and *r*_13_, did not (Fig. 3b, right panel). The same $a_i^{opt}$ values yielded zero values for the connections *r*_12_ and *r*_23_ for both choices of module outputs (see Supplementary material, section 5.1). We conclude that the inferred connections, *r*_12_ and *r*_23_, are merely retroactive and are induced solely by protein sequestration, while the interactions described by connection coefficients, *r*_21_, *r*_32_, and *r*_13_, are regulatory connections. Thus, in case of a regulatory feedback connection between modules, which are not linked by immediate feedforward connections, our approach correctly distinguishes between regulatory and sequestration connections. Sets of permissible parameters for unique network reconstruction include both the total protein abundances and numerous kinetic constants (see Supplementary material, section 5.1).

More complex regulatory feedback mechanisms are found for a three-tiered RAF/MEK/ERK cascade, which is evolutionary conserved in eukaryotic cells. Active ERK (module 3) binds to active CRAF and BRAF monomers (module 1) and inhibits their kinase activities by phosphorylation of inhibitory sites.^37,38^ A complete mechanistic description considers homo- and hetero CRAF and BRAF dimers and includes numerous reaction steps.^39^ Using a simplified mechanistic description of this regulatory feedback, Supplementary material (section 5) shows that selecting communicating species as weighted sums of the free phosphorylated proteins and inter-modular protein complexes, precise discrimination between sequestration and regulatory feedbacks and unique network reconstruction can also be achieved.

We next considered a regulatory feedback loop between two cascade tiers that are connected through immediate feedforward activation (Fig. 3c). Here module 2 activates module 3, whereas module 3 output routes back into module 2, as a regulatory feedback loop. Because module 3 can also retroactively affect module 2 through sequestration, we can ask how effects of sequestration compete with regulatory feedback. To simplify the analysis, we again used a standard Michaelis-Menten description of the regulatory feedback. Assuming that an active form (*pX*_3_) of the tier 3 enzyme activates or inhibits the formation of the productive complex $\left[ {pX_1 \cdot \cdot X_2} \right]$ in a non-competitive manner, the reaction rate of *X*_2_ activation was multiplied by the following multiplier, (1 + *u*_2_[*pX*_3_]/*k*_10_)/(1 + [*pX*_3_]/*k*_10_).^36^ This regulatory feedback is positive, if *u*_2_ > 1, and it is negative, if 0 < *u*_2_ < 1.

As above, we defined communicating species by Eqs. (18) and (19) and monitored the signs of connection coefficients *r*_*ij*_ when changing the weight parameters *a*_*i*_. The signs of connection coefficients depend on the signs of the global response coefficients (Eq. 7), which in turn depend on the changes in the concentrations of free active enzymes and enzyme-substrate complexes (components of communicating species) caused by parameter perturbations. Instructively, upon perturbations to the protein abundance $(X_3^{tot})$ of module 3, regulatory and sequestration connections affected the concentrations $\left[ {pX_2 \cdot \cdot X_3} \right]$ and [*pX*_2_] in different ways. When $X_3^{tot}$ is perturbed, regulatory feedback loops decreased or increased both these concentrations together, whereas sequestration (i.e., retroactive) connections changed $\left[ {pX_2 \cdot \cdot X_3} \right]$ and [*pX*_2_] in opposite directions. For example, if $X_3^{tot}$ decreases, negative regulatory feedback increases and positive regulatory feedback decreases both $\left[ {pX_2 \cdot \cdot X_3} \right]$ and [*pX*_2_], whereas sequestration effects decrease $\left[ {pX_2 \cdot \cdot X_3} \right]$ and increase [*pX*_2_].

As a result, when both regulatory feedback loop and sequestration feedback connection are present, following perturbations to $X_3^{tot}$, the concentrations $\left[ {pX_2 \cdot \cdot X_3} \right]$ and [*pX*_2_] change either in concert or in opposite ways, depending on the relative strengths of these two feedback interactions. If a regulatory feedback dominates (when *u*_2_ is greater than a certain threshold value), both $\left[ {pX_2 \cdot \cdot X_3} \right]$ and [*pX*_2_] move in the same direction. In this case, the coefficients *r*_12_ and *r*_13_ change their sign at certain values $a_i = a_i^{opt}$, suggesting solely sequestration connections (Fig. 3c, right panel). However, the coefficients, *r*_21_, *r*_32_, and *r*_23_, do not change their signs, indicating regulatory feedforward activation connections (*r*_21_ and *r*_32_) and positive regulatory feedback from module 3 to module 2 (*r*_23_). In other words, at $a_i = a_i^{opt}$ the connection matrix *r*_*ij*_ displays zero values for *r*_12_ and *r*_13_ and non-zero values for *r*_21_, *r*_32_, and *r*_23_ (Fig. 3c, the connection matrix for a strong regulatory feedback, *u*_2_ = 50.5).

However, when sequestration effects dominate (when *u*_2_is smaller than a threshold value), the concentrations $\left[ {pX_2 \cdot \cdot X_3} \right]$ and [*pX*_2_] change in opposite directions upon perturbations to $X_3^{tot}$. Then, the connection coefficient *r*_23_ also changes the sign (together with the other sequestration connections, *r*_12_ and *r*_13_) at the $a_i = a_i^{opt}$ values, whereas the connection coefficients, *r*_21_ and *r*_32_, remain non-zero (Fig. 3c, the connection matrix for a weak regulatory feedback, *u*_2_ = 1.75).

Supplementary Table 11 illustrates similar results for a negative regulatory feedback. When this feedback dominates, the coefficient *r*_23_ does not change the sign for different weight parameters *a*_*i*_, suggesting a regulatory feedback, whereas the connections that appear as a result of solely enzyme sequestrations (*r*_12_ and *r*_13_) change their signs. However, when a negative regulatory feedback is weak and enzyme sequestration dominates, the sequestration feedback forces the coefficient *r*_23_ to change its sign with changes in the weights *a*_*i*_.

## Discussion

A computational method, termed Modular Response Analysis (MRA), allows reconstructing direct causative connections in intracellular signaling networks from measured responses of an entire network to systematic perturbations.^7,28^ However, MRA, as any method for solving reverse engineering problems, suffers from several limitations. One weak point is the instability of solutions with respect to noise in the input data.^9^ Fortunately, numerous statistical re-formulations of MRA, including Maximum likelihood (ML), Monte Carlo-ML and Bayesian variants of MRA^4,23–25,27,29,30^ have successfully addressed this problem for practical applications of MRA to noisy and incomplete data (as a recent review see ref. ^31^).

The other limitation of MRA is related to enzyme sequestrations in protein modification reactions (also known as retroactivity or inter-modular mass transfer).^7,15,16,40^ This problem of mass transfer has been known for a long time and also discussed in the original MRA paper,^7^ yet it still challenges signaling network reconstruction.^41–43^ Recently, Prabakaran and colleagues have highlighted this challenge for MRA by inferring surprisingly different network circuitries, depending on which protein abundances in the network were perturbed.^20^

In the present work, we conclude that findings of different network circuitries using distinct sets of perturbations are explained by the violation of the modular insulation condition (see Eqs. 6 and 11). This key MRA condition is commonly violated when the concentrations of inter-modular complexes are of the same order of magnitude as the conserved abundance of a protein participating in an inter-modular complex (which is formed, for instance, by an enzyme from one module and its substrate from the other module). Experimentally, the concentration of the inter-modular complex and the conserved abundance of a protein in this complex can be compared using co-immunoprecipitation of an enzyme and its substrate and comparing the western blot intensity with the intensity for enzyme or substrate concentration in the leftover lysate, using the same blot. The modular insulation condition^7^ did not hold in the experiments of Prabakaran and colleagues due to considerable sequestration of active MEK (ppMEK) by its substrate ERK, which belongs to a module downstream of the MEK module.^20^ Because enzyme sequestration and resulting retroactivity is often observed in cell signaling networks, it is imperative to extend MRA-based reconstruction methods to networks with protein complexes bridging different modules.

Here we show that additional measurements allow us to computationally restore the modular insulation condition for a range of network topologies, including those used in the experiments of Prabakaran and colleagues.^20^ This permits a unique network reconstruction for different selections of applied perturbations, including all conserved protein abundances and a range of kinetic constants. A key to our approach is an alternative definition of communicating species (solely for computational network reconstruction purposes), as weighted combinations of free active enzymes and enzyme-substrate complexes that bridge different network modules. Provided that global responses to perturbations of free active enzymes and enzyme-substrate complexes can be separately measured, we have computationally reconstructed connection coefficients (*r*_*ij*_) of direct, causative interactions between network modules for different values of weights (*a*_*i*_). We considered kinase cascades (ubiquitous for cell signaling) and first reconstructed cascades with no regulatory feedback loops and cascades where regulatory feedback loops connect modules that are not linked by immediate feedforward connections. We demonstrated that sequestration-induced connections (i.e. retroactivity) are distinguished from regulatory connections by computationally determining the weight parameter values (termed $a_i^{opt}$) that simultaneously nullify all sequestration-induced connections. We also showed that at these $a_i^{opt}$ values the modular insulation condition (Eq. 6) is restored. Thus, for these network circuitries, we were able to uniquely reconstruct networks and reveal the mechanistic nature of direct, causative connections.

Current biochemical techniques allow us to measure the concentrations of both free active enzymes and inter-modular protein complexes. For instance, co-immunoprecipitation of an enzyme and its substrate from the other module will determine the concentration of an inter-modular protein complex, whereas immunoprecipitation using an antibody against phosphorylated enzyme will determine the active enzyme concentration. Importantly, only the relative concentration changes are detected using Western blotting, but our approach performs equally well when the input data are relative changes in the concentration of proteins and protein complexes. This is explained by the fact that both global responses and local connection coefficients can be determined in terms of either absolute changes^7,28^ or relative changes, defined by the logarithmic derivatives in Eqs. (4) and (5).^7^ Therefore, the perturbation-induced global responses of module outputs can be readily analyzed in terms of the relative changes in the concentrations of proteins and protein complexes (see Supplementary material, section 3).

When cascade modules are connected by both regulatory and sequestration feedbacks, MRA infers a dominant feedback. In particular, a regulatory feedback will manifest itself if its strength exceeds a certain threshold, whereas for weaker feedback strengths only a sequestration feedback will be revealed. For different and more complex network topologies this approach may also have a limited applicability, requiring more prior information. For instance, for inhibitory cascades additional knowledge about which of two proteins in an inter-modular complex is an enzyme and which is a substrate is required (see Supplementary material, section 6). Importantly, this knowledge can be obtained from consensus phosphorylation sequences for many kinases and enzyme-substrate databases.^44–48^ For signaling networks where a module operates as a hub activating several downstream modules (see Supplementary material, sections 7 and 8), our approach is capable of inferring retroactivity feedback loops. However, for these networks, not all sequestration-induced connections can be nullified at a single set of $a_i = a_i^{opt}$, because they change their signs at the alternative weight coefficient sets. As a result, the inferred connections can differ for alternative perturbations. Sections 7 and 8 in Supplementary material demonstrate that for different sets of perturbation parameters, sequestration connections exhibit the greatest variability. Therefore, minimization of the sum of squares of sequestration-induced connections minimizes the discrepancy between the inferred connection matrices, improving the network inference quality. Importantly, all inferred regulatory connections are qualitatively similar, when the sum of squares of the sequestration-induced connection coefficients is minimized (see Supplementary material, sections 7 and 8). Because a network reconstruction process can be concurrently impaired by both protein sequestration effects and noise, we have also checked that the use of the new communicating species (module outputs) suggested by our approach does not significantly decrease accuracy and precision of MRA-based network reconstruction^49^ (see Supplementary material, section 9).

Crosstalk between pathways often operates as feedforward and feedback regulatory loops mediated by protein (de)phosphorylation.^50^ Yet, these regulatory interactions are not the only biological mechanisms of pathway crosstalk. Protein sequestration in competing protein-protein interactions is a key mechanism that regulates crosstalk between the Hippo and RAS/RAF/MEK/ERK pathways.^51,52^ In this and similar cases, our approach correctly identifies sequestration connections, which also play regulatory roles. Yet, similarly as for a hub network topology, sequestration-induced connections cannot be nullified at a single set of weight parameters, $a_i = a_i^{opt}$. Consequently, the selection of module outputs, which we have computationally explored, does not restore the modular insulation condition.

In summary, our approach significantly extends MRA-based methods to cover a range signaling networks with considerable reactivity interactions between modules. At the cost of additional measurements, this approach computationally restores the modular insulation condition and permits unmistakable network reconstruction for a range of signaling motifs and experimental perturbations.

## Methods

All numerical simulations were carried out in R^53^ using the package dMod^54^ and its dependencies and custom functions. The magnitude of parameter perturbations used in calculations was 10% expect in the calculations that explored noise in the data (Section 9 in Supplementary material), where the perturbation magnitude was 50%. Plots were generated with the package ggplot2^55^ which is part of the collection of packages called tidyverse. The scripts (file “Code.tar.gz”) are available as supplementary information for numerical results in the main text. Analytical calculations were partly done using Mathematica^56^ and Sage^57^ software packages.

## Supplementary information

Supplemental material

The comprehenisve set of R scripts

The sets of permissible parameters related to Supplemental Equation 30

Sage worksheet that illustrates restoration of modular insulation condition for 2-tier inhibitory cascade (Supplemental material, section 6)

## Data Availability

All data generated or analysed during this study are included in this published article (and its supplementary information files).

## Supplementary information

**Supplementary Information** accompanies the paper on the *npj Systems Biology and Applications* website (10.1038/s41540-019-0096-1).

## Code availability

The comprehensive set of scripts written in R (file “Code.tar.gz”) is available for download as supplementary information.
