# Supplementary material for: Mapping connections in signaling networks with ambiguous modularity
Source: NPJ Syst Biol Appl. 2019 May 23;5:19. doi: 10.1038/s41540-019-0096-1 (PMC6533310; doi:10.1038/s41540-019-0096-1)
Supplement: Supplementary file 1 — Supplemental material [file 41540_2019_96_MOESM1_ESM.docx]

**Mapping connections in signaling networks with ambiguous modularity**

**Supplementary material**

Daniel Lill^1,2+^, Oleksii S. Rukhlenko^2+^, Anthony James Mc Elwee^2^, Eugene Kashdan^2,3^, Jens Timmer^1,4^, Boris N. Kholodenko^2,5,6,7*^

1 – Institute of Physics, University of Freiburg, Freiburg, Germany

2 – Systems Biology Ireland, University College Dublin, Dublin, Ireland

3 – School of Mathematics and Statistics, University College Dublin, Ireland

4 – BIOSS Centre for Biological Signaling Studies, University of Freiburg, Freiburg, Germany

5 – Conway Institute of Biomolecular & Biomedical Research, University College Dublin, Dublin, Ireland

6 – School of Medicine and Medical Science, University College Dublin, Dublin, Ireland

7 – Department of Pharmacology, Yale University School of Medicine, New Haven, CT, USA

^+^ – equal contribution

^*^ – correspondence: [boris.kholodenko@ucd.ie](mailto:boris.kholodenko@ucd.ie)

# Section 1. Enzyme sequestration can violate modular insulation condition

Consider a simple model with enzyme ($X$) in module *i* which phosphorylates a substrate ($Y$) in module *j*.

| $X\to pX$ | $k_{1}$ | (1). |
| --- | --- | --- |
| $pX\to X$ | $k_{2}$ |  |
| $pX+Y\leftrightarrow pX\text{∙∙}Y$ | $K_{d}=k_{-3}/k_{3}$ |  |
| $pX\text{∙∙}Y\to pX+pY$ | $k_{4}$ |  |
| $pY\to Y$ | $k_{5}$ |  |

The temporal dynamics of these species from two interacting modules are described by the following system of equations (brackets denote concentrations),

| $d\left[ X \right]/dt=-k_{1}\left[ X \right]+k_{2}\left[ pX \right]$ | (2). |
| --- | --- |
| $d\left[ pX \right]/dt=k_{1}\left[ X \right]-k_{2}\left[ pX \right]-k_{3}\left[ pX \right]\left[ Y \right]+\left( k_{-3}+k_{4} \right)\left[ pX_{i}\text{∙∙}Y_{j} \right]$ |  |
| $d\left[ pX\text{∙∙}Y \right]/dt=k_{3}\left[ pX \right]\left[ Y \right]-\left( k_{-3}+k_{4} \right)\left[ pX\text{∙∙}Y \right]$ |  |
| $d\left[ Y \right]/dt=-k_{3}\left[ pX \right]\left[ Y \right]+k_{5}\left[ pY \right]$ |  |
| $d\left[ pY \right]/dt=k_{4}\left[ pX\text{∙∙}Y \right]-k_{5}\left[ pY \right]$ |  |

This ODE system has the following two first integrals that correspond to moiety conservations of $X$ and $Y$,

| $\left[ X \right]+\left[ pX \right]+\left[ pX\text{∙∙}Y \right]=X^{tot}$ | (3). |
| --- | --- |
| $\left[ Y \right]+\left[ pY \right]+\left[ pX\text{∙∙}Y \right]=Y^{tot}$ |  |

Here $X^{tot}$ and $Y^{tot}$ are the total abundances of the enzyme and the substrate.

Using these moiety conservations, free, inactive forms, $\left[ X \right]$ and $\left[ Y \right],$ can be expressed in terms of 3 linearly independent concentrations, $\left[ pX \right]$, $\left[ pX\text{∙∙}Y \right]$ and $\left[ pY \right]$. The steady-state behavior of these species is described as follows (Supplementary Equation 4),

| $d\left[ pX \right]/dt=f_{pX}=k_{1}\left( X^{tot}-\left[ pX \right]-\left[ pX\text{∙∙}Y \right] \right)-k_{2}\left[ pX \right]-$  $-k_{3}\left[ pX \right]\left( Y^{tot}-\left[ pY \right]-\left[ pX\text{∙∙}Y \right] \right)+\left( k_{-3}+k_{4} \right)\left[ pX\text{∙∙}Y \right]$ | (4). |
| --- | --- |
| $d\left[ pX\text{∙∙}Y \right]/dt=f_{pXY}=k_{3}\left[ pX_{i} \right]\left( Y^{tot}-\left[ pY \right]-\left[ pX\text{∙∙}Y \right] \right)-\left( k_{-3}+k_{4} \right)\left[ pX\text{∙∙}Y \right]$ |  |
| $d\left[ pY \right]/dt=f_{pY}=k_{4}\left[ pX\text{∙∙}Y \right]-k_{5}\left[ pY \right]$ |  |

Given $pX$ and $pY$ are selected as communicating species, the connection coefficients can be derived from MRA equations (Eq. 7), as follows,

| $r_{ji}=R_{j X^{tot}}/R_{i X^{tot}}=\frac{\partial\left[ pY \right]}{\partial X^{tot}}/\frac{\partial\left[ pX \right]}{\partial X^{tot}}$ | (5). |
| --- | --- |
| $r_{ij}=R_{i Y^{tot}}/R_{j Y^{tot}}=\frac{\partial\left[ pX \right]}{\partial Y^{tot}}/\frac{\partial\left[ pY \right]}{\partial Y^{tot}}$ |  |

To calculate the global responses ($R_{iX^{tot}}$ and $R_{iY^{tot}}$) of the communicating species, we have to determine the steady state derivatives of three independent steady state concentrations, $\left[ pX \right]$, $\left[ pX\text{∙∙}Y \right]$ and $\left[ pY \right]$, with respect to the total enzyme ($X^{tot}$) and substrate ($Y^{tot}$) abundances. The derivatives of these steady state solutions are expressed, as follows,

| $\left( \begin{aligned} \begin{matrix} \frac{\partial\left[ pX \right]}{\partial X^{tot}} \\ \frac{\partial\left[ pX\text{∙∙}Y \right]}{\partial X^{tot}} \end{matrix} \\ \frac{\partial\left[ pY \right]}{\partial X^{tot}} \end{aligned} \right)=-J^{-1}\left( \begin{aligned} \begin{matrix} \frac{\partial f_{pX}}{\partial X^{tot}} \\ \frac{\partial f_{pXY}}{\partial X^{tot}} \end{matrix} \\ \frac{\partial f_{pY}}{\partial X^{tot}} \end{aligned} \right)=\left( \begin{aligned} \begin{matrix} k_{1}\frac{k_{5}\left( k_{4}+k_{-3} \right)+k_{3}\left( k_{4}+k_{5} \right)\left[ pX \right]}{g} \\ \frac{k_{1}k_{3}k_{5}\left[ Y \right]}{g} \end{matrix} \\ \frac{k_{1}k_{3}k_{4}\left[ Y \right]}{g} \end{aligned} \right)$  $\left( \begin{aligned} \begin{matrix} \frac{\partial\left[ pX \right]}{\partial Y^{tot}} \\ \frac{\partial\left[ pX\text{∙∙}Y \right]}{\partial Y^{tot}} \end{matrix} \\ \frac{\partial\left[ pY \right]}{\partial Y^{tot}} \end{aligned} \right)=-J^{-1}\left( \begin{aligned} \begin{matrix} \frac{\partial f_{pX}}{\partial Y^{tot}} \\ \frac{\partial f_{pXY}}{\partial Y^{tot}} \end{matrix} \\ \frac{\partial f_{pY}}{\partial Y^{tot}} \end{aligned} \right)=\left( \begin{aligned} \begin{matrix} -\frac{k_{1}k_{3}k_{5}}{g} \\ \frac{\left( k_{1}+k_{2} \right)k_{3}k_{5}}{g} \end{matrix} \\ \frac{\left( k_{1}+k_{2} \right)k_{3}k_{4}}{g} \end{aligned} \right)\left[ pX \right]$  $g=k_{1}k_{3}k_{5}\left[ Y \right]+\left( k_{1}+k_{2} \right)\left( k_{5}\left( k_{-3}+k_{4} \right)+k_{3}\left( k_{4}+k_{5} \right)\left[ pX \right] \right)>0$ | (6). |
| --- | --- |

Here $J$ is the Jacobian of the system in Supplementary Equation 4.

Then the connection coefficient $r_{ij}$ representing connection strength from module *j* to module *i* can be expressed as follows

| $r_{ij}=\frac{\partial\left[ pX \right]}{\partial Y^{tot}}/\frac{\partial\left[ pY \right]}{\partial Y^{tot}}=-\frac{k_{1}k_{5}}{\left( k_{1}+k_{2} \right)k_{4}}<0$ | (7). |
| --- | --- |

Supplementary Equation 7 shows that $r_{ij}$ is less than zero, which would correspond to a negative feedback.

However, as we know species $Y$ does not enzymatically modify species $X$, and therefore the negative value of $r_{ij}$ accounts for purely protein sequestration. Importantly, following perturbation to $Y^{tot}$, the signs of the derivatives of the steady state concentrations of free active enzyme ($\partial\left[ pX \right]/\partial Y^{tot}$) and the enzyme-substrate complex ($\partial\left[ pX\text{∙∙}Y \right]/\partial Y^{tot}$) with respect to $Y^{tot}$ ) are opposite, while following perturbation to $X^{tot}$ these signs are both positive (Supplementary Equations 5 and 6). Therefore, if we select a weighted sum of the free enzyme and the complex ($\left[ pX \right]+a\text{∙[}pX\text{∙∙}Y]$) as a communicating species of the enzyme module, at some value ($a^{opt}$) of the weight parameter ($a$) the derivative, $\partial\left( \left[ pX \right]+a^{opt}\text{∙}\left[ pX\text{∙∙}Y \right] \right)/\partial Y^{tot}=\left( -k_{1}k_{3}k_{5}+a^{opt}\left( k_{1}+k_{2} \right)k_{3}k_{5} \right)\left[ pX \right]/g$, will become zero, yielding zero $r_{ij}$, while $r_{ji}$ will remain positive. From Supplementary Equations 5 and 6 it follows that if $a^{opt}=k_{1}/\left( k_{1}+k_{2} \right)$ the communicating species $\left[ pX \right]+a^{opt}\text{∙[}pX\text{∙∙}Y]$ will not be directly perturbed by the changes in the $Y$ abundance, $Y^{tot}$. Therefore, the modular insulation condition (Eqs. 6 and 11) will hold for modules *i* and *j* and perturbations to the protein abundances, $X^{tot}$ and $Y^{tot}$. This simple example illustrates a general idea of the proposed approach, i.e. a weighted sum of the responses to a perturbation in the protein abundance ($Y^{tot}$) of free enzyme form and the inter-modular complex can nullify the sequestration-induced connection coefficient ($r_{ij}$).

The case when enzyme $X$ inactivates protein $Y$ by phosphorylation on the inhibitory sites can be analyzed similarly as above. In this case non-phosphorylated $Y$ is an active form, selected as a communicating species, and the optimal value of weight parameter ($a_{opt}$) will be the same, equal to $k_{1}/\left( k_{1}+k_{2} \right)$.

# Section 2. Model of MEK-ERK cascade

## 2.1. Analytical investigation of the model of MEK-ERK cascade

To obtain the equations that govern the steady state behavior of communicating species $x_{1}^{a}$ and $x_{2}^{a}$, we first introduce new variables for the ODE system (Eq. 8 of the main text) as linear combinations of the old variables. This can be done using a linear transformation matrix ($M$),

| $\left( \begin{matrix} \begin{matrix} [MEK] \\ [ppMEK] \end{matrix} \\ \left[ ppMEK\text{∙∙}ERK \right] \\ \begin{matrix} \left[ ERK \right] \\ [pERK] \\ \begin{matrix} [pERK\text{∙∙}PTP] \\ [PTP] \end{matrix} \end{matrix} \end{matrix} \right)\to\left( \begin{matrix} \begin{matrix} [MEK] \\ X_{a}^{*}=\left[ ppMEK \right]+a [ppMEK\text{∙∙}ERK] \end{matrix} \\ \left[ ppMEK\text{∙∙}ERK \right] \\ \begin{matrix} [ERK] \\ Y_{a}^{*}=\left[ pERK \right]+[pERK\text{∙∙}PTP] \\ \begin{matrix} [pERK\text{∙∙}PTP] \\ [PTP] \end{matrix} \end{matrix} \end{matrix} \right)=M(a)\left( \begin{matrix} \begin{matrix} [MEK] \\ [ppMEK] \end{matrix} \\ \left[ ppMEK\text{∙∙}ERK \right] \\ \begin{matrix} \left[ ERK \right] \\ [pERK] \\ \begin{matrix} [pERK\text{∙∙}PTP] \\ [PTP] \end{matrix} \end{matrix} \end{matrix} \right)$ | (8). |
| --- | --- |

In Supplementary Equation 8 $M$ is given by the following expression

| $M=\left( \begin{matrix} 1 & 0 & 0 & 0 & 0 & 0 & 0 \\ 0 & 1 & \boldsymbol{a} & 0 & 0 & 0 & 0 \\ 0 & 0 & 1 & 0 & 0 & 0 & 0 \\ 0 & 0 & 0 & 1 & 0 & 0 & 0 \\ 0 & 0 & 0 & 0 & 1 & 1 & 0 \\ 0 & 0 & 0 & 0 & 0 & 1 & 0 \\ 0 & 0 & 0 & 0 & 0 & 0 & 1 \end{matrix} \right)$ | (9). |
| --- | --- |

The ODE system (Eq. 8) transforms as follows,

| $\boldsymbol{f}\left( \boldsymbol{x} \right)\to\boldsymbol{f}^{'}\left( \boldsymbol{x}^{'} \right)=M\boldsymbol{f}(M^{-1}\boldsymbol{x}')$ | (10). |
| --- | --- |

Then, we apply moiety conservations (Eq. 8, Supplementary Equations 11 and 12). We choose to express the dynamics of non-active forms of MEK and ERK, and free form of PTP, in terms of their active forms and protein-protein complexes.

| $\left[ MEK \right]=MEK^{tot} -\left[ ppMEK \right]-\left[ ppMEK\text{∙∙}ERK \right]$ | (11). |
| --- | --- |
| $\left[ ERK \right]=ERK^{tot}-\left[ ppMEK\text{∙∙}ERK \right]-\left[ pERK \right]-\left[ pERK\text{∙∙}PTP \right]$ |  |
| $\left[ PTP \right]=PTP^{tot}-\left[ pERK\text{∙∙}PTP \right]$ |  |

| $\frac{d}{dt}\left( \begin{aligned} \begin{aligned} \begin{aligned} \begin{matrix} \begin{matrix} \left[ MEK \right] \\ x_{1}^{a} \end{matrix} \\ \left[ ppMEK\text{∙∙}ERK \right] \\ \left[ ERK \right] \end{matrix} \\ x_{2}^{a} \end{aligned} \\ \left[ pERK\text{∙∙}PTP \right] \end{aligned} \\ \left[ PTP \right] \end{aligned} \right)=\boldsymbol{f}'\left( \boldsymbol{x}' \right)\underset{\Rightarrow}{\begin{matrix} moiety \\ conservation \end{matrix}}$  $\frac{d}{dt}\left( \begin{matrix} \begin{matrix} x_{1}^{a} \\ \left[ ppMEK\text{∙∙}ERK \right] \end{matrix} \\ x_{2}^{a} \\ \left[ pERK\text{∙∙}PTP \right] \end{matrix} \right)=\boldsymbol{g}\left( X_{a}^{*},\left[ ppMEK\text{∙∙}ERK \right],Y_{a}^{*},\left[ pERK\text{∙∙}PTP \right] \right)$ | (12). |
| --- | --- |

After that we exploit quasi-steady state approximation to eliminate the concentrations of the protein-protein complexes, which are not communicating species themselves

| $\frac{d}{dt}\left( \begin{matrix} \left[ ppMEK\text{∙∙}ERK \right] \\ \left[ pERK\text{∙∙}PTP \right] \end{matrix} \right)=0\Rightarrow\frac{d}{dt}\left( \begin{matrix} x_{1}^{a} \\ x_{2}^{a} \end{matrix} \right)=\boldsymbol{g}^{'}\left( x_{1}^{a},x_{2}^{a} \right)$ | (13). |
| --- | --- |

Applying the procedure described above (Supplementary Equation 8-13), the equations governing dynamics of communicating species $x_{1}^{a}$ and $x_{2}^{a}$ are given by,

$$f_{1}=\frac{1}{2ak_{3}^{on}}\left( ak_{2}\left( k_{3}^{off}+ak_{3}^{on}\left( ERK^{tot}-x_{2}^{a} \right)+k_{4}-k_{3}^{on}x_{1}^{a}-\sqrt{\left( k_{3}^{off}+ak_{3}^{on}\left( ERK^{tot}-x_{2}^{a} \right)+k_{4} \right)^{2}+2k_{3}^{on}\left( k_{3}^{off}+ak_{3}^{on}\left( x_{2}^{a}-ERK^{tot} \right)+ k_{4} \right)x_{1}^{a}+{k_{3}^{on}}^{2}{x_{1}^{a}}^{2}} \right)-k_{1}\left( k_{3}^{off}-ak_{3}^{off}+a^{2}k_{3}^{on}\left( x_{2}^{a}-ERK^{tot} \right)+k_{4}+k_{3}^{on}x_{1}^{a}-\sqrt{\left( k_{3}^{off}+ak_{3}^{on}\left( ERK^{tot}-x_{2}^{a} \right)+k_{4} \right)^{2}+2k_{3}^{on}\left( k_{3}^{off}+ak_{3}^{on}\left( x_{2}^{a}-ERK^{tot} \right)+k_{4} \right)x_{1}^{a}+{k_{3}^{on}}^{2}{x_{1}^{a}}^{2}}+a\left( \sqrt{\left( k_{3}^{off}+ak_{3}^{on}\left( ERK^{tot}-x_{2}^{a} \right)+k_{4} \right)^{2}+2k_{3}^{on}\left( k_{3}^{off}+ak_{3}^{on}\left( x_{2}^{a}-ERK^{tot} \right)+k_{4} \right)x_{1}^{a}+{k_{3}^{on}}^{2}{x_{1}^{a}}^{2}}-k_{4}+ k_{3}^{on}\left( -x_{2}^{a}+ERK^{tot}+x_{1}^{a}-2MEK^{tot} \right) \right) \right) \right)$$

(14).

$f_{2}=\frac{1}{2ak_{3}^{on}}\left( k_{4}\left( k_{3}^{off}-ak_{3}^{on}x_{2}^{a}+ak_{3}^{on}ERK^{tot}+k_{4}+k_{3}^{on}x_{1}^{a}-\sqrt{4a{k_{3}^{on}}^{2}\left( x_{2}^{a}-ERK^{tot} \right)x_{1}^{a} +\left( k_{3}^{off}+ak_{3}^{on}\left( ERK^{tot}-x_{2}^{a} \right)+k_{4}+k_{3}^{on}x_{1}^{a} \right)^{2}} \right) \right)-\left( k_{6}\left( k_{5}^{off}+k_{5}^{on}x_{2}^{a}+k_{6}+k_{5}^{on}PTP^{tot}-\frac{1}{k_{5}^{on}}\sqrt{\left( k_{5}^{off}+k_{6}+k_{5}^{on}\left( x_{2}^{a}+PTP^{tot} \right) \right)^{2}-4{k_{5}^{on}}^{2}x_{2}^{a}PTP^{tot}} \right) \right)$

As can be seen from Supplementary Equation 14, $x_{1}^{a}$ depends on $ERK^{tot}$ and other parameters from the ERK-module. We next want to find the exact value of free parameter $a=a^{opt}$ such that the modular insulation condition is fulfilled for perturbation of $ERK^{tot}$

| $\frac{\partial x_{1}^{a}}{\partial ERK^{tot}}\left( a^{opt} \right)=0$ | (15). |
| --- | --- |

The solution of this equation is the following

| $a^{opt} =\frac{k_{1}}{k_{1}+k_{2}}$ | (16). |
| --- | --- |

Given $a=a^{opt}$, the equations governing communicating species are the following

$$f_{1}^{a^{opt}}=k_{1}{MEK}^{tot}-\left( \left( k_{1}+k_{2} \right)x_{1}^{a^{opt}} \right)$$

(17).

$$f_{2}^{a^{opt}}=\frac{1}{2}\left( \frac{1}{k_{1}k_{3}^{on}}\left( k_{4}\left( k_{1}+k_{2} \right)\left( k_{3}^{off}-\frac{k_{1}k_{3}^{on}x_{2}^{a^{opt}}}{k_{1}+k_{2}}+\frac{{k_{1}k_{3}^{on}ERK}^{tot}}{k_{1}+k_{2}}+k_{4}+k_{3}^{on}x_{1}^{a^{opt}}-\left[ \frac{1}{\left( k_{1}+k_{2} \right)^{2}}4k_{1}{k_{3}^{on}}^{2}\left( x_{2}^{a^{opt}}-ERK^{tot} \right)\left( k_{1}+k_{2} \right)x_{1}^{a^{opt}}+\left( k_{2}\left( k_{3}^{off}+k_{4}+k_{3}^{on}x_{1}^{a^{opt}} \right)+k_{1}\left( k_{3}^{off}+k_{4}+k_{3}^{on}\left( ERK^{tot}-x_{2}^{a^{opt}}+x_{1}^{a^{opt}} \right) \right) \right)^{2} \right] \right) \right)-\frac{k_{6}}{k_{5}^{on}}\left( k_{5}^{off}+k_{5}^{on}x_{2}^{a^{opt}}+k_{6}+k_{5}^{on}PTP^{tot}-\sqrt{-4x_{2}^{a^{opt}}{k_{5}^{on}}^{2}PTP^{tot}+\left( k_{5}^{off}+k_{6}+k_{5}^{on}\left( x_{2}^{a^{opt}}+PTP^{tot} \right) \right)^{2}} \right) \right)$$

It can be seen that $x_{1}^{a^{opt}}$ is now independent of all parameters intrinsic to the ERK-module. Given communicating species defined as in Eq. 12 (see main text) and $a=a^{opt}$, MRA yields the following local response (i.e., connection coefficient) matrix (model parameter values are presented in Supplementary Table 1), which is invariant to a wide range of perturbations of ERK module (permissible parameters are: $ERK^{tot}$, $PTP^{tot}$, $k_{3}^{on}$, $k_{3}^{off}$, $k_{4}$, $k_{5}^{on}$, $k_{5}^{off}$ and $k_{6}$) given that MEK module was perturbed by changing $MEK^{tot}$

| $\boldsymbol{r}=\left( \begin{matrix} -1 & 0 \\ 1.52 & -1 \end{matrix} \right)$ | (18). |
| --- | --- |

The analysis of the case of communicating species defined as in Eq. 15 can be done similarly. In particular, the solution of Supplementary Equation 15 yields the same value of $a^{opt}$ (see Eq. 15), and the local response matrix at $a=a^{opt}$ is uniquely reconstructed from the same spectrum of perturbations. The numerical values of local response matrix elements (model parameter values are presented in Supplementary Table 1) are the following:

| $\boldsymbol{r}=\left( \begin{matrix} -1 & 0 \\ 1.48 & -1 \end{matrix} \right)$  Note that these elements are only slightly differ from the connection coefficients in Supplementary Equation 18. | (19). |
| --- | --- |

## 2.2 Parameter values for the model of MEK-ERK cascade

The parameter values used for the model of MEK-ERK cascade were previously published in [^1^](#_ENREF_1)

| **Supplementary Table 1. Parameter values for the model of MEK-ERK cascade** | |
| --- | --- |
| $MEK^{tot}$ | 100 nM |
| $ERK^{tot}$ | 1000 nM |
| $PTP^{tot}$ | 100 nM |
| $k_{1}$ | 0.1 s^-1^ |
| $k_{2}$ | 0.1 s^-1^ |
| $k_{3}^{on}$ | 0.01 nM^-1^s^-1^ |
| $k_{3}^{off}$ | 1.0 s^-1^ |
| $k_{4}$ | 1.0 s^-1^ |
| $k_{6}^{on}$ | 0.01 nM^-1^s^-1^ |
| $k_{6}^{off}$ | 1 s^-1^ |
| $k_{5}$ | 1.0 s^-1^ |

# Section 3. Applying the approach when the input data are relative changes in the concentration of proteins and protein complexes

The definitions of communicating species according to Eqs. 12, 15 and 18-21 in the main text presume that the absolute values of the concentration of proteins and protein complexes are experimentally measured. While such measurements are possible, many widely used experimental techniques (e.g., Western Blot) allow only the relative concentration changes to be measured. Here, we show how relative concentrations can be used to obtain, for any non-negative weight parameters $\vec{a}$, the global response matrix to infer the matrices of connection coefficients, using our approach.

Similarly to Supplementary material Section 1, consider a case when an enzyme ($X_{i}$) in module *i* phosphorylates a substrate ($Y_{j}$) in module *j*. $X_{i}$ and $Y_{j}$ can be communicating species or internal species. Assume that free active species $\left[ pX_{i} \right]$ of module *i* and inter-modular complex $\left[ pX_{i}\text{∙∙}Y_{j} \right]$ are measured in terms of the absolute concentrations. Before a perturbation, these concentrations are denoted by $\left[ pX_{i} \right]^{0}$ and $\left[ pX_{i}\text{∙∙}Y_{j} \right]^{0}$, whereas following the a perturbation, for instance to module *k*, these concentrations are denoted by and $\left[ pX_{i}\text{∙∙}Y_{j} \right]^{k}$. The corresponding element of the global response matrix is then found using the central fractional differences [^2^](#_ENREF_2):

| $R_{ij}\left( a \right)= 2\frac{\left[ pX \right]^{k}+a\left[ pX\text{∙∙}Y \right]^{k}-\left( \left[ pX \right]^{0}+a\left[ pX\text{∙∙}Y \right]^{0} \right)}{\left[ pX \right]^{k}+a\left[ pX\text{∙∙}Y \right]^{k}+\left[ pX \right]^{0}+a\left[ pX\text{∙∙}Y \right]^{0}}=2\frac{\left[ pX \right]^{k}-\left[ pX \right]^{0}+a\left( \left[ pX\text{∙∙}Y \right]^{k}-\left[ pX\text{∙∙}Y \right]^{0} \right)}{\left[ pX \right]^{k}+\left[ pX \right]^{0}+a(\left[ pX\text{∙∙}Y \right]^{k}+\left[ pX\text{∙∙}Y \right]^{0})}$ | (20) |
| --- | --- |

Widely used experimental techniques can only determine the fractional changes of $\left[ pX_{i} \right]$ and $\left[ pX_{i}\text{∙∙}Y_{j} \right]$ taken separately. Therefore, we will define the global responses $R_{ij}^{r}$ for relative concentration changes as follows,

| $R_{ij}^{r}\left( a_{r} \right)=2\frac{\left[ pX \right]^{k}-\left[ pX \right]^{0}}{\left[ pX \right]^{k}+\left[ pX \right]^{0}}+2a_{r}\cdot\frac{\left[ pX\text{∙∙}Y \right]^{k}-\left[ pX\text{∙∙}Y \right]^{0}}{\left[ pX\text{∙∙}Y \right]^{k}+\left[ pX\text{∙∙}Y \right]^{0}}$ | (21) |
| --- | --- |

Supplementary Equations 20 and 21 are equivalent up to scaling factor given the following substitutions

| $a_{r}=a\cdot\frac{\left[ pX\text{∙∙}Y \right]^{k}+\left[ pX\text{∙∙}Y \right]^{0}}{\left[ pX \right]^{k}+\left[ pX \right]^{0}}$  $\gamma=\frac{1}{1+a_{r}}$  $R_{ij}\left( a \right)=\gamma{\cdot R}_{ij}^{r}\left( a_{r} \right)$ | (22) |
| --- | --- |

Since the MRA equations (Eq. 7) are scale invariant [^3^](#_ENREF_3), formulations of global response matrices according to Supplementary Equations 20 and 21 will lead to identical network topologies.

Numerical investigations confirm our analytical proof that connection matrices reconstructed using formulations of global response matrices in Supplementary Equations 20 and 21 possess the same properties (including distinguishing between sequestration and regulatory connections and invariance of connection matrix for wide range of perturbations). Particularly, in cases when a set $\vec{a}=\vec{a}^{opt}$ exists for the global response matrix defined in terms of absolute concentration (Supplementary Equation 20), the set $\vec{a}_{r}=\vec{a}_{r}^{opt}$ also exists for the global response matrix defined for relative concentrations (Supplementary Equation 21). This set $\vec{a}_{r}=\vec{a}_{r}^{opt}$ nullifies the sequestration-induced connections and yields the connection matrix invariant with respect to different perturbations for the global response matrix. For example, the connection matrix reconstructed from global response matrix defined as in Supplementary Equation 21 at $\vec{a}_{r}=\vec{a}_{r}^{opt}$ for MEK-ERK cascade model (see Eq. 8 and Supplementary Table 1) is the following:

| $r_{ij}=\left( \begin{matrix} -1 & 0 \\ 0.97 & -1 \end{matrix} \right)$ | (23). |
| --- | --- |

The set of permissible parameters is: 1)$MEK^{tot}$ for MEK module and 2) $ERK^{tot}$, $PTP^{tot}$, $k_{3}^{on}$, $k_{3}^{off}$, $k_{4}$, $k_{5}^{on}$, $k_{5}^{off}$ and $k_{6}$ for ERK module.

# Section 4. A model of a three-tier cascade with no regulatory feedback connections

| **Supplementary Table 2. Kinetic equations of the model of 3-tier cascade without feedback connections** | |
| --- | --- |
| $d\left[ X_{1} \right]/dt$ | $-v_{1}\left[ X_{1} \right]/\left( k_{1}+\left[ X_{1} \right] \right)+v_{2}\left[ pX_{1} \right]/\left( k_{2}+\left[ pX_{1} \right] \right)$ |
| $d\left[ pX_{1} \right]/dt$ | $v_{1}\left[ X_{1} \right]/\left( k_{1}+\left[ X_{1} \right] \right)-v_{2}\left[ pX_{1} \right]/\left( k_{2}+\left[ pX_{1} \right] \right)-k_{31}\left[ X_{2} \right]\left[ pX_{1} \right]+k_{32}\left[ pX_{1}\text{∙∙}X_{2} \right]+k_{4}\left[ pX_{1}\text{∙∙}X_{2} \right]$ |
| $d\left[ pX_{1}\text{∙∙}X_{2} \right]/dt$ | $k_{31}\left[ X_{2} \right]\left[ pX_{1} \right]-k_{32}\left[ pX_{1}\text{∙∙}X_{2} \right]-k_{4}\left[ pX_{1}\text{∙∙}X_{2} \right]$ |
| $d\left[ X_{2} \right]/dt$ | $-k_{31}\left[ X_{2} \right]\left[ pX_{1} \right]+k_{32}\left[ pX_{1}\text{∙∙}X_{2} \right]+v_{5}\left[ pX_{2} \right]/\left( k_{5}+\left[ pX_{2} \right] \right)$ |
| $d\left[ pX_{2} \right]/dt$ | $k_{4}\left[ pX_{1}\text{∙∙}X_{2} \right]-v_{5}\left[ pX_{2} \right]/\left( k_{5}+\left[ pX_{2} \right] \right)-k_{61}\left[ X_{3} \right]\left[ pX_{2} \right]+k_{62}\left[ pX_{2}\text{∙∙}X_{3} \right]+k_{7}\left[ pX_{2}\text{∙∙}X_{3} \right]$ |
| $d\left[ pX_{2}\text{∙∙}X_{3} \right]/dt$ | $k_{61}\left[ X_{3} \right]\left[ pX_{2} \right]-k_{62}\left[ pX_{2}\text{∙∙}X_{3} \right]-k_{7}\left[ pX_{2}\text{∙∙}X_{3} \right]$ |
| $d\left[ X_{3} \right]/dt$ | $-k_{61}\left[ X_{3} \right]\left[ pX_{2} \right]+k_{62}\left[ pX_{2}\text{∙∙}X_{3} \right]+v_{8}\left[ pX_{3} \right]/\left( k_{8}+\left[ pX_{3} \right] \right)$ |
| $d\left[ pX_{3} \right]/dt$ | $k_{7}\left[ pX_{2}\text{∙∙}X_{3} \right]-v_{8}\left[ pX_{3} \right]/\left( k_{8}+\left[ pX_{3} \right] \right)$ |
| Moiety conservation relations | $X_{1}^{tot}=\left[ X_{1} \right]+\left[ pX_{1} \right]+\left[ pX_{1}\text{∙∙}X_{2} \right]$  $X_{2}^{tot}=\left[ X_{2} \right]+\left[ pX_{2} \right]+\left[ pX_{1}\text{∙∙}X_{2} \right]+\left[ pX_{2}\text{∙∙}X_{3} \right]$  $X_{3}^{tot}=\left[ X_{3} \right]+\left[ pX_{3} \right]+\left[ pX_{2}\text{∙∙}X_{3} \right]$ |

| **Supplementary Table 3. Parameter values of the model of a three-tier cascade without feedback connections** | |
| --- | --- |
| $X_{1}^{tot}$ | 100 nM |
| $X_{2}^{tot}$ | 100 nM |
| $X_{3}^{tot}$ | 100 nM |
| $k_{1}$ | 100 nM |
| $v_{1}$ | 1.5 nM/s |
| $k_{2}$ | 100 nM |
| $v_{2}$ | 1 nM/s |
| $k_{31}$ | 0.01 nM^-1^s^-1^ |
| $k_{32}$ | 0.4 s^-1^ |
| $k_{4}$ | 0.05 s^-1^ |
| $k_{5}$ | 100 nM |
| $v_{5}$ | 5 nM/s |
| $k_{61}$ | 0.05 nM^-1^s^-1^ |
| $k_{62}$ | 0.4 s^-1^ |
| $k_{7}$ | 08 s^-1^ |
| $k_{8}$ | 100 nM |
| $v_{8}$ | 1 nM/s |

Perturbing permissible parameters (see the main text and below), obtaining the global response matrix (Eq. 5) for the communicating species, presented in Eq. 18, and solving Eqs. 7, the connection matrix $r_{ij}$is calculated as a function of the weight parameters, $\vec{a}=\{a_{i}\}$. At $\vec{a}=\vec{a}^{opt}$, we find that connections $r_{12}$, $r_{23}$ and $r_{13}$ are equal to zero

| $r_{ij}=\left( \begin{matrix} -1 & 0 & 0 \\ 0.23 & -1 & 0 \\ 0 & 0.28 & -1 \end{matrix} \right)$ | (24). |
| --- | --- |

The corresponding set of permissible parameters are: 1) $X^{tot}$, $k_{1}$, $v_{1}$, $k_{2}$ and $v_{2}$ for module 1, 2) $Y^{tot}$, $k_{31}$, $k_{32}$, $k_{4}$, $k_{5}$ and $v_{5}$ for module 2 and 3) $Z^{tot}$, $k_{61}$, $k_{62}$, $k_{7}$, $k_{8}$ or $v_{8}$ for module 3.

Next we show that connection matrix can be uniquely reconstructed only for specifically defined communicating species and by perturbing permissible parameters. In the table below we show that for standard choice of communicating species, corresponding to free active kinase concentrations ($\vec{a}=0$) the reconstructed connection matrices are different for different parameters perturbed, but for communicating species corresponding to optimal weights ($\vec{a}=\vec{a}^{opt}$) the reconstructed connection matrices are identical.

| **Supplementary Table 4. Reconstructed connection matrices for different sets of perturbed parameters.** | | |
| --- | --- | --- |
| Perturbed parameters | Standard choice of communicating species (free active kinase concentrations, $r(\overset{⃗}{a}=0)$) | Communicating species corresponding to optimal weights $(r(\overset{⃗}{a}=a^{opt})$) |
| $X^{tot}$, $Y^{tot}$, $Z^{tot}$ | $\left( \begin{matrix} -1.00 & -0.19 & 0.01 \\ 0.33 & -1.00 & -0.49 \\ -0.00 & 0.18 & -1.00 \end{matrix} \right)$ | $\left( \begin{matrix} -1.00 & -0.00 & -0.00 \\ 0.23 & -1.00 & 0.00 \\ -0.00 & 0.28 & -1.00 \end{matrix} \right)$ |
| $k_{2}$, $k_{32}$, $Z^{tot}$ | $\left( \begin{matrix} -1.00 & -0.21 & 0.00 \\ 0.32 & -1.00 & -0.49 \\ 0.00 & 0.16 & -1.00 \end{matrix} \right)$ | $\left( \begin{matrix} -1.00 & -0.00 & -0.00 \\ 0.23 & -1.00 & 0.00 \\ -0.00 & 0.28 & -1.00 \end{matrix} \right)$ |
| $X^{tot}$, $Y^{tot}$, $k_{8}$ | $\left( \begin{matrix} -1.00 & -0.19 & -0.01 \\ 0.28 & -1.00 & 0.54 \\ -0.00 & 0.18 & -1.00 \end{matrix} \right)$ | $\left( \begin{matrix} -1.00 & -0.00 & -0.00 \\ 0.23 & -1.00 & 0.00 \\ -0.00 & 0.28 & -1.00 \end{matrix} \right)$ |
| $v_{2}$, $Y^{tot}$, $k_{8}$ | $\left( \begin{matrix} -1.00 & -0.19 & -0.01 \\ 0.26 & -1.00 & 0.54 \\ -0.00 & 0.18 & -1.00 \end{matrix} \right)$ | $\left( \begin{matrix} -1.00 & -0.00 & -0.00 \\ 0.23 & -1.00 & 0.00 \\ -0.00 & 0.28 & -1.00 \end{matrix} \right)$ |
| $k_{1}$, $k_{32}$, $v_{8}$ | $\left( \begin{matrix} -1.00 & -0.21 & -0.00 \\ 0.26 & -1.00 & 0.57 \\ 0.00 & 0.16 & -1.00 \end{matrix} \right)$ | $\left( \begin{matrix} -1.00 & -0.00 & -0.00 \\ 0.23 & -1.00 & 0.00 \\ -0.00 & 0.28 & -1.00 \end{matrix} \right)$ |
| $v_{1}$, $v_{5}$, $k_{61}$ | $\left( \begin{matrix} -1.00 & 0.15 & 0.17 \\ 0.32 & -1.00 & -0.51 \\ 0.00 & 0.16 & -1.00 \end{matrix} \right)$ | $\left( \begin{matrix} -1.00 & -0.00 & -0.00 \\ 0.23 & -1.00 & 0.00 \\ -0.00 & 0.28 & -1.00 \end{matrix} \right)$ |
| $k_{1}$, $k_{5}$, $k_{62}$ | $\left( \begin{matrix} -1.00 & 0.15 & 0.17 \\ 0.31 & -1.00 & -0.51 \\ -0.00 & 0.17 & -1.00 \end{matrix} \right)$ | $\left( \begin{matrix} -1.00 & -0.00 & -0.00 \\ 0.23 & -1.00 & 0.00 \\ -0.00 & 0.28 & -1.00 \end{matrix} \right)$ |
| $v_{2}$, $k_{32}$, $k_{62}$ | $\left( \begin{matrix} -1.00 & -0.21 & -0.00 \\ 0.31 & -1.00 & -0.51 \\ 0.00 & 0.16 & -1.00 \end{matrix} \right)$ | $\left( \begin{matrix} -1.00 & -0.00 & -0.00 \\ 0.23 & -1.00 & 0.00 \\ -0.00 & 0.28 & -1.00 \end{matrix} \right)$ |
| $X^{tot}$, $k_{31}$, $k_{7}$ | $\left( \begin{matrix} -1.00 & -0.20 & 0.00 \\ 0.28 & -1.00 & 0.54 \\ 0.00 & 0.17 & -1.00 \end{matrix} \right)$ | $\left( \begin{matrix} -1.00 & -0.00 & -0.00 \\ 0.23 & -1.00 & 0.00 \\ -0.00 & 0.28 & -1.00 \end{matrix} \right)$ |
| $k_{1}$, $k_{31}$, $k_{7}$ | $\left( \begin{matrix} -1.00 & -0.20 & 0.00 \\ 0.26 & -1.00 & 0.54 \\ -0.00 & 0.17 & -1.00 \end{matrix} \right)$ | $\left( \begin{matrix} -1.00 & -0.00 & -0.00 \\ 0.23 & -1.00 & 0.00 \\ -0.00 & 0.28 & -1.00 \end{matrix} \right)$ |

Applying a similar procedure for the communicating species defined by Eq. 19, the following connection matrix was obtained at optimal weight parameters $\vec{a}=\vec{a}^{opt}$:

| $r_{ij}=\left( \begin{matrix} -1 & 0 & 0 \\ 0.22 & -1 & 0 \\ 0 & 0.26 & -1 \end{matrix} \right)$ | (25). |
| --- | --- |

The corresponding set of permissible parameters are: 1) $X_{1}^{tot}$, $k_{1}$, $v_{1}$, $k_{2}$ and $v_{2}$ for module 1, 2) $X_{2}^{tot}$, $k_{31}$, $k_{32}$ for module 2 and 3) $X_{3}^{tot}$, $k_{61}$, $k_{62}$ for module 3.

# Section 5. Models of three-tier cascades with regulatory feedback connections

## 5.1 Model of a three-tier cascade with a feedback connection from module 3 to module 1

| **Supplementary Table 5. Kinetic equations of the model of a three-tier cascade with feedback connection from module 3 to module 1** | |
| --- | --- |
| $d\left[ X_{1} \right]/dt$ | $-v_{1}\left[ X_{1} \right]/\left( k_{1}+\left[ X_{1} \right] \right)\cdot\left( 1+u_{1}\left[ pX_{3} \right]/k_{9} \right)/\left( 1+\left[ pX_{3} \right]/k_{9} \right)+v_{2}\left[ pX_{1} \right]/\left( k_{2}+\left[ pX_{1} \right] \right)$ |
| $d\left[ pX_{1} \right]/dt$ | $v_{1}\left[ X_{1} \right]/\left( k_{1}+\left[ X_{1} \right] \right)\cdot\left( 1+u_{1}\left[ pX_{3} \right]/k_{9} \right)/\left( 1+\left[ pX_{3} \right]/k_{9} \right)-v_{2}\left[ pX_{1} \right]/\left( k_{2}+\left[ pX_{1} \right] \right)-k_{31}\left[ X_{2} \right]\left[ pX_{1} \right]+k_{32}\left[ pX_{1}\text{∙∙}X_{2} \right]+k_{4}\left[ pX_{1}\text{∙∙}X_{2} \right]$ |
| $d\left[ pX_{1}\text{∙∙}X_{2} \right]/dt$ | $k_{31}\left[ X_{2} \right]\left[ pX_{1} \right]-k_{32}\left[ pX_{1}\text{∙∙}X_{2} \right]-k_{4}\left[ pX_{1}\text{∙∙}X_{2} \right]$ |
| $d\left[ X_{2} \right]/dt$ | $-k_{31}\left[ X_{2} \right]\left[ pX_{1} \right]+k_{32}\left[ pX_{1}\text{∙∙}X_{2} \right]+v_{5}\left[ pX_{2} \right]/\left( k_{5}+\left[ pX_{2} \right] \right)$ |
| $d\left[ pX_{2} \right]/dt$ | $k_{4}\left[ pX_{1}\text{∙∙}X_{2} \right]-v_{5}\left[ pX_{2} \right]/\left( k_{5}+\left[ pX_{2} \right] \right)-k_{61}\left[ X_{3} \right]\left[ pX_{2} \right]+k_{62}\left[ pX_{2}\text{∙∙}X_{3} \right]+k_{7}\left[ pX_{2}\text{∙∙}X_{3} \right]$ |
| $d\left[ pX_{2}\text{∙∙}X_{3} \right]/dt$ | $k_{61}\left[ X_{3} \right]\left[ pX_{2} \right]-k_{62}\left[ pX_{2}\text{∙∙}X_{3} \right]-k_{7}\left[ pX_{2}\text{∙∙}X_{3} \right]$ |
| $d\left[ X_{3} \right]/dt$ | $-k_{61}\left[ X_{3} \right]\left[ pX_{2} \right]+k_{62}\left[ pX_{2}\text{∙∙}X_{3} \right]+v_{8}\left[ pX_{3} \right]/\left( k_{8}+\left[ pX_{3} \right] \right)$ |
| $d\left[ pX_{3} \right]/dt$ | $k_{7}\left[ pX_{2}\text{∙∙}X_{3} \right]-v_{8}\left[ pX_{3} \right]/\left( k_{8}+\left[ pX_{3} \right] \right)$ |
| Moiety conservation relations | $X_{1}^{tot}=\left[ X_{1} \right]+\left[ pX_{1} \right]+\left[ pX_{1}\text{∙∙}X_{2} \right]$  $X_{2}^{tot}=\left[ X_{2} \right]+\left[ pX_{2} \right]+\left[ pX_{1}\text{∙∙}X_{2} \right]+\left[ pX_{2}\text{∙∙}X_{3} \right]$  $X_{3}^{tot}=\left[ X_{3} \right]+\left[ pX_{3} \right]+\left[ pX_{2}\text{∙∙}X_{3} \right]$ |

| **Supplementary Table 6. Parameter values of the model of a three-tier cascade with feedback connection from module 3 to module 1** | |
| --- | --- |
| $X_{1}^{tot}$ | 100 nM |
| $X_{2}^{tot}$ | 100 nM |
| $X_{3}^{tot}$ | 100 nM |
| $k_{1}$ | 100 nM |
| $v_{1}$ | 1.5 nM/s |
| $k_{2}$ | 100 nM |
| $v_{2}$ | 1 nM/s |
| $k_{31}$ | 0.002 nM^-1^s^-1^ |
| $k_{32}$ | 0.4 s^-1^ |
| $k_{4}$ | 0.05 s^-1^ |
| $k_{5}$ | 100 nM |
| $v_{5}$ | 5 nM/s |
| $k_{61}$ | 0.005 nM^-1^s^-1^ |
| $k_{62}$ | 0.4 s^-1^ |
| $k_{7}$ | 0.05 s^-1^ |
| $k_{8}$ | 100 nM |
| $v_{8}$ | 1 nM/s |
| $k_{9}$ | 50 nM |
| $u_{1}$ | 0.5 |

Perturbing permissible parameters, obtaining the global response matrix (Eq. 5) for the communicating species, presented in Eq. 18, and solving Eqs. 7, we calculate the connection matrix $r_{ij}$ as a function of the weight parameters, $\vec{a}=\{a_{i}\}$. At $\vec{a}=\vec{a}^{opt}$ we find that the connection matrix is the following,

| $r_{ij}=\left( \begin{matrix} -1.00 & 0.00 & -0.09 \\ 0.77 & -1.00 & 0.00 \\ -0.00 & 0.53 & -1.00 \end{matrix} \right)$ | (26). |
| --- | --- |

The corresponding set of permissible parameters are: 1) $X^{tot}$, $k_{1}$, $v_{1}$, $k_{2}$ and $v_{2}$ for module 1, 2) $Y^{tot}$, $k_{31}$, $k_{32}$, $k_{4}$, $k_{5}$ and $v_{5}$ for module 2 and 3) $Z^{tot}$, $k_{61}$, $k_{62}$, $k_{7}$, $k_{8}$ or $v_{8}$ for module 3.

Applying a similar procedure for the communicating species defined by Eq. 19, we obtain the following connection matrix at optimal weight parameters, $\vec{a}=\vec{a}^{opt}$,

| $r_{ij}=\left( \begin{matrix} -1.00 & 0.00 & -0.10 \\ 0.74 & -1.00 & 0.00 \\ -0.00 & 0.54 & -1.00 \end{matrix} \right)$ | (27). |
| --- | --- |

The corresponding set of permissible parameters are: 1) $X_{1}^{tot}$, $k_{1}$, $v_{1}$, $k_{2}$ and $v_{2}$ for module 1, 2) $X_{2}^{tot}$, $k_{31}$, $k_{32}$ for module 2 and 3) $X_{3}^{tot}$, $k_{61}$, $k_{62}$ for module 3.

## 5.2 A three-tier cascade with negative feedback from module 3 to module 1. The feedback is mechanistically modelled and involves a protein-protein complex formation

| **Supplementary Table 7. Kinetic equations of the model of a three-tier cascade with negative feedback that is mechanistically modeled** | |
| --- | --- |
| $d\left[ X_{1} \right]/dt$ | $-v_{1}\left[ X_{1} \right]/\left( k_{1}+\left[ X_{1} \right] \right)+v_{2}\left[ pX_{1} \right]/\left( k_{2}+\left[ pX_{1} \right] \right)+k_{12}\left[ pX_{1}\text{∙∙}pX_{2} \right]$ |
| $d\left[ pX_{1} \right]/dt$ | $v_{1}\left[ X_{1} \right]/\left( k_{1}+\left[ X_{1} \right] \right)-v_{2}\left[ pX_{1} \right]/\left( k_{2}+\left[ pX_{1} \right] \right)-k_{31}\left[ X_{2} \right]\left[ pX_{1} \right]+k_{32}\left[ pX_{1}\text{∙∙}X_{2} \right]+k_{4}\left[ pX_{1}\text{∙∙}X_{2} \right]-k_{111}\left[ pX_{1} \right]\left[ pX_{3} \right]+k_{112}\left[ pX_{1}\text{∙∙}pX_{3} \right]$ |
| $d\left[ pX_{1}\text{∙∙}X_{2} \right]/dt$ | $k_{31}\left[ X_{2} \right]\left[ pX_{1} \right]-k_{32}\left[ pX_{1}\text{∙∙}X_{2} \right]-k_{4}\left[ pX_{1}\text{∙∙}X_{2} \right]$ |
| $d\left[ X_{2} \right]/dt$ | $-k_{31}\left[ X_{2} \right]\left[ pX_{1} \right]+k_{32}\left[ pX_{1}\text{∙∙}X_{2} \right]+v_{5}\left[ pX_{2} \right]/\left( k_{5}+\left[ pX_{2} \right] \right)$ |
| $d\left[ pX_{2} \right]/dt$ | $k_{4}\left[ pX_{1}\text{∙∙}X_{2} \right]-v_{5}\left[ pX_{2} \right]/\left( k_{5}+\left[ pX_{2} \right] \right)-k_{61}\left[ X_{3} \right]\left[ pX_{2} \right]+k_{62}\left[ pX_{2}\text{∙∙}X_{3} \right]+k_{7}\left[ pX_{2}\text{∙∙}X_{3} \right]$ |
| $d\left[ pX_{2}\text{∙∙}X_{3} \right]/dt$ | $k_{61}\left[ X_{3} \right]\left[ pX_{2} \right]-k_{62}\left[ pX_{2}\text{∙∙}X_{3} \right]-k_{7}\left[ pX_{2}\text{∙∙}X_{3} \right]$ |
| $d\left[ X_{3} \right]/dt$ | $-k_{61}\left[ X_{3} \right]\left[ pX_{2} \right]+k_{62}\left[ pX_{2}\text{∙∙}X_{3} \right]+v_{8}\left[ pX_{3} \right]/\left( k_{8}+\left[ pX_{3} \right] \right)$ |
| $d\left[ pX_{3} \right]/dt$ | $k_{7}\left[ pX_{2}\text{∙∙}X_{3} \right]-v_{8}\left[ pX_{3} \right]/\left( k_{8}+\left[ pX_{3} \right] \right)-k_{111}\left[ pX_{1} \right]\left[ pX_{3} \right]+k_{112}\left[ pX_{1}\text{∙∙}pX_{3} \right]+k_{12}\left[ pX_{1}\text{∙∙}pX_{3} \right]$ |
| $d\left[ pX_{1}\text{∙∙}pX_{3} \right]/dt$ | $k_{111}\left[ pX_{1} \right]\left[ pX_{3} \right]-k_{112}\left[ pX_{1}\text{∙∙}pX_{3} \right]-k_{12}\left[ pX_{1}\text{∙∙}pX_{3} \right]$ |
| Moiety conservation relations | $X_{1}^{tot}=\left[ X_{1} \right]+\left[ pX_{1} \right]+\left[ pX_{1}\text{∙∙}X_{2} \right]+\left[ pX_{1}\text{∙∙}pX_{3} \right]$  $X_{2}^{tot}=\left[ X_{2} \right]+\left[ pX_{2} \right]+\left[ pX_{1}\text{∙∙}X_{2} \right]+\left[ pX_{2}\text{∙∙}X_{3} \right]$  $X_{3}^{tot}=\left[ X_{3} \right]+\left[ pX_{3} \right]+\left[ pX_{2}\text{∙∙}X_{3} \right]+\left[ pX_{1}\text{∙∙}pX_{3} \right]$ |

| **Supplementary Table 8. Parameter values for** **the model of a three-tier cascade with negative feedback that is mechanistically modeled** | |
| --- | --- |
| $X_{1}^{tot}$ | 100 nM |
| $X_{2}^{tot}$ | 100 nM |
| $X_{3}^{tot}$ | 100 nM |
| $k_{1}$ | 100 nM |
| $v_{1}$ | 1.5 nM/s |
| $k_{2}$ | 100 nM |
| $v_{2}$ | 1 nM/s |
| $k_{31}$ | 0.2 nM^-1^s^-1^ |
| $k_{32}$ | 0.4 s^-1^ |
| $k_{4}$ | 0.05 s^-1^ |
| $k_{5}$ | 100 nM |
| $v_{5}$ | 5 nM/s |
| $k_{61}$ | 0.5 nM^-1^s^-1^ |
| $k_{62}$ | 0.4 s^-1^ |
| $k_{7}$ | 0.05 s^-1^ |
| $k_{8}$ | 100 nM |
| $v_{8}$ | 1 nM/s |
| $k_{111}$ | 0.2 nM^-1^s^-1^ |
| $k_{112}$ | 0.4 s^-1^ |
| $k_{12}$ | 0.05 s^-1^ |

The choice of communicating species is the following

| $\begin{matrix} x_{1}^{a}=\left[ pX_{1} \right]+a_{1}\cdot\left[ pX_{1}\text{∙∙}X_{2} \right] \\ x_{2}^{a}=\left[ pX_{2} \right]+a_{2}\cdot\left[ pX_{2}\text{∙∙}X_{3} \right] \\ x_{3}^{a}=\left[ pX_{3} \right]+a_{3}\cdot\left[ pX_{3}\text{∙∙}pX_{1} \right] \end{matrix}$ | (28) |
| --- | --- |

| $\begin{matrix} x_{1}^{a}=\left[ pX_{1} \right]+a_{1}\cdot\left[ pX_{1}\text{∙∙}X_{2} \right]+a_{3}\cdot\left[ pX_{3}\text{∙∙}pX_{1} \right] \\ x_{2}^{a}=\left[ pX_{2} \right]+a_{1}\cdot\left[ pX_{1}\text{∙∙}X_{2} \right]+a_{2}\cdot\left[ pX_{2}\text{∙∙}X_{3} \right] \\ x_{3}^{a}=[pX_{3}]+a_{2}\cdot\left[ pX_{2}\text{∙∙}X_{3} \right]+a_{3}\cdot\left[ pX_{3}\text{∙∙}pX_{1} \right] \end{matrix}$ | (29) |
| --- | --- |

Perturbing permissible parameters, obtaining the global response matrix (Eq. 5) for the communicating species, presented in Supplementary Equation 28, and solving Eqs. 7, the connection matrix $r_{ij}$ is calculated as a function of the weight parameters, $\vec{a}=\{a_{i}\}$. At $\vec{a}=\vec{a}^{opt}$, the connection matrix found is the following,

| $r_{ij}=\left( \begin{matrix} -1 & 0 & -0.42 \\ 0.82 & -1 & 0 \\ 0 & 0.59 & -1 \end{matrix} \right)$ | (30). |
| --- | --- |

The corresponding set of permissible parameters are: 1) $X_{1}^{tot}$, $k_{1}$, $v_{1}$,$k_{2}$, $v_{2}$, $k_{111}$, $k_{112}$ and $k_{12}$ for module 1, 2) $X_{2}^{tot}$, $k_{31}$, $k_{32}$, $k_{4}$, $k_{5}$ and $v_{5}$ for module 2 and 3) $X_{3}^{tot}$, $k_{61}$, $k_{62}$, $k_{7}$, $k_{8}$ or $v_{8}$ for module 3.

Applying a similar procedure for the communicating species defined by Supplementary Equation 29, we obtain the following connection matrix at optimal weight parameters, $\vec{a}=\vec{a}^{opt}$

| $r_{ij}=\left( \begin{matrix} -1 & 0 & -0.35 \\ 0.74 & -1 & 0 \\ 0 & 0.63 & -1 \end{matrix} \right)$ | (31). |
| --- | --- |

The corresponding permissible combinations of perturbed parameters are presented in SI, file “permissible_perturbations.csv”.

## 5.3 Model of a three-tier cascade with a regulatory feedback loop between two tiers that are connected through an immediate feedforward activation

| **Supplementary Table 9. Kinetic equations of the model of a three-tier cascade with a regulatory feedback from module 3 to module 2 and sequestration connection between the same modules 2 and 3** | |
| --- | --- |
| $d\left[ X_{1} \right]/dt$ | $-v_{1}\left[ X_{1} \right]/\left( k_{1}+\left[ X_{1} \right] \right)+v_{2}\left[ pX_{1} \right]/\left( k_{2}+\left[ pX_{1} \right] \right)$ |
| $d\left[ pX_{1} \right]/dt$ | $v_{1}\left[ X_{1} \right]/\left( k_{1}+\left[ X_{1} \right] \right)-v_{2}\left[ pX_{1} \right]/\left( k_{2}+\left[ pX_{1} \right] \right)-k_{31}\left[ pX_{2} \right]\left[ pX_{1} \right]\cdot\left( 1+u_{2}\left[ pX_{3} \right]/k_{10} \right)/\left( 1+\left[ pX_{3} \right]/k_{10} \right)+k_{32}\left[ pX_{1}\text{∙∙}X_{2} \right]+k_{4}\left[ pX_{1}\text{∙∙}X_{2} \right]$ |
| $d\left[ pX_{1}\text{∙∙}X_{2} \right]/dt$ | $k_{31}\left[ pX_{2} \right]\left[ pX_{1} \right]\cdot\left( 1+u_{2}\left[ pX_{3} \right]/k_{10} \right)/\left( 1+\left[ pX_{3} \right]/k_{10} \right)-k_{32}\left[ pX_{1}\text{∙∙}X_{2} \right]-k_{4}\left[ pX_{1}\text{∙∙}X_{2} \right]$ |
| $d\left[ X_{2} \right]/dt$ | $-k_{31}\left[ X_{2} \right]\left[ pX_{1} \right] \left( 1+u_{2}\left[ pX_{3} \right]/k_{10} \right)/\left( 1+\left[ pX_{3} \right]/k_{10} \right)+k_{32}\left[ pX_{1}\text{∙∙}X_{2} \right]+v_{5}\left[ pX_{2} \right]/\left( k_{5}+\left[ pX_{2} \right] \right)$ |
| $d\left[ pX_{2} \right]/dt$ | $k_{4}\left[ pX_{1}\text{∙∙}X_{2} \right]-v_{5}\left[ pX_{2} \right]/\left( k_{5}+\left[ pX_{2} \right] \right)-k_{61}\left[ X_{3} \right]\left[ pX_{2} \right]+k_{62}\left[ pX_{2}\text{∙∙}X_{3} \right]+k_{7}\left[ pX_{2}\text{∙∙}X_{3} \right]$ |
| $d\left[ pX_{2}\text{∙∙}X_{3} \right]/dt$ | $k_{61}\left[ X_{3} \right]\left[ pX_{2} \right]-k_{62}\left[ pX_{2}\text{∙∙}X_{3} \right]-k_{7}\left[ pX_{2}\text{∙∙}X_{3} \right]$ |
| $d\left[ X_{3} \right]/dt$ | $-k_{61}\left[ X_{3} \right]\left[ pX_{2} \right]+k_{62}\left[ pX_{2}\text{∙∙}X_{3} \right]+v_{8}\left[ pX_{3} \right]/\left( k_{8}+\left[ pX_{3} \right] \right)$ |
| $d\left[ pX_{3} \right]/dt$ | $k_{7}\left[ pX_{2}\text{∙∙}X_{3} \right]-v_{8}\left[ pX_{3} \right]/\left( k_{8}+\left[ pX_{3} \right] \right)$ |
| Moiety conservation relations | $X_{1}^{tot}=\left[ X_{1} \right]+\left[ pX_{1} \right]+\left[ pX_{1}\text{∙∙}X_{2} \right]$  $X_{2}^{tot}=\left[ X_{2} \right]+\left[ pX_{2} \right]+\left[ pX_{1}\text{∙∙}X_{2} \right]+\left[ pX_{2}\text{∙∙}X_{3} \right]$  $X_{3}^{tot}=\left[ X_{3} \right]+\left[ pX_{3} \right]+\left[ pX_{2}\text{∙∙}X_{3} \right]$ |

| **Supplementary Table 10. Parameter values of the model of a three-tier cascade with a regulatory feedback and sequestration connection between the same modules** | |
| --- | --- |
| $X_{1}^{tot}$ | 100 nM |
| $X_{2}^{tot}$ | 100 nM |
| $X_{3}^{tot}$ | 100 nM |
| $k_{1}$ | 100 nM |
| $v_{1}$ | 1.5 nM/s |
| $k_{2}$ | 100 nM |
| $v_{2}$ | 1 nM/s |
| $k_{31}$ | 0.2 nM^-1^s^-1^ |
| $k_{32}$ | 0.4 s^-1^ |
| $k_{4}$ | 0.05 s^-1^ |
| $k_{5}$ | 100 nM |
| $v_{5}$ | 5 nM/s |
| $k_{61}$ | 0.5 nM^-1^s^-1^ |
| $k_{62}$ | 0.4 s^-1^ |
| $k_{7}$ | 0.05 s^-1^ |
| $k_{8}$ | 100 nM |
| $v_{8}$ | 1 nM/s |
| $k_{10}$ | 50 nM |
| $u_{2}$ | [0;100] |

**Supplementary Table 11. Reconstructed connection matrices** $\boldsymbol{r}_{\boldsymbol{ij}}$ **for different strengths (**$\boldsymbol{u}_{\boldsymbol{2}}$**) of the negative regulatory feedback and optimal weights** $\boldsymbol{a}_{\boldsymbol{i}}^{\boldsymbol{opt}}$ **for a three-tier cascade model shown in Supplementary Tables 9 and 10. The matrix elements that correspond to retroactive (i.e. sequestration) connections are depicted in red. For network reconstruction, the total protein abundances,** $\boldsymbol{X}_{\boldsymbol{1}}^{\boldsymbol{tot}}$**,** $\boldsymbol{X}_{\boldsymbol{2}}^{\boldsymbol{tot}}$ **and** $\boldsymbol{X}_{\boldsymbol{3}}^{\boldsymbol{tot}}$**, were perturbed.**

| Strong regulatory feedback |  |  |  |
| --- | --- | --- | --- |
| Matrix $r_{ij}$  $u_{2}=0.01$  $\vec{a}=\vec{a}^{opt}$; | -1.00 | 0.00 | 0.00 |
|  | 0.69 | -1.00 | -0.16 |
|  | 0.00 | 0.70 | -1.00 |
| Weak regulatory feedback |  |  |  |
| Matrix $r_{ij}$  $u_{2}=0.1$  $\vec{a}=\vec{a}^{opt}$ | -1.00 | 0.00 | 0.00 |
|  | 0.58 | -1.00 | 0.00 |
|  | 0.00 | 0.75 | -1.00 |

# Section 6. Model of a two-tier inhibitory cascade

We now consider a different, simple example of a two-tier signaling cascade, where a communicating signaling protein of the first tier inhibits a communicating protein of the second tier. The enzyme in the first module can be a kinase that phosphorylates a downstream kinase on inhibitory sites, or a phosphatase that dephosphorylates the kinase activating sites in module 2 (Supplementary Figure 1A, Supplementary Tables 12 and 13). An active enzyme (*pX*_1_) of module 1 makes a complex with its substrate, an active enzyme (*pX*_2_) of module 2. This complex bridges both modules. Similar to the above, the complex concentration $\left[ pX_{1}\text{∙∙}pX_{2} \right]$ is included in the moiety conservation ($X_{1}^{tot}$, $X_{2}^{tot}$) for each module of the cascade. When active *pX*_1_ and *pX*_2_ are selected as module outputs, the module insulation condition (Eq. 6) is violated for perturbation to either total abundance. Therefore, different perturbation sets, such as two total abundances ($X_{1}^{tot}$, $X_{2}^{tot}$) or the total abundance of one protein and a kinetic constant, may lead to reconstructions of different network circuitries. Therefore, we again consider a weighted sum of the free active protein and protein-protein complex concentrations either for only first module (Supplementary Equation 32), or to the second module (Supplementary Equation 33), or for both modules (Supplementary Equation 34),

| $X_{1}^{a}=\left[ pX_{1} \right]+a\left[ pX_{1}\text{∙∙}pX_{2} \right]$; $X_{2}^{a}=\left[ pX_{2} \right]$ | (32) |
| --- | --- |

| $X_{1}^{a}=\left[ pX_{1} \right]; X_{2}^{a}=\left[ pX_{2} \right]+a\left[ pX_{1}\text{∙∙}pX_{2} \right]$ | (33) |
| --- | --- |

| $X_{1}^{a}=\left[ pX_{1} \right]+a\left[ pX_{1}\text{∙∙}pX_{2} \right]; X_{2}^{a}=\left[ pX_{2} \right]+a\left[ pX_{1}\text{∙∙}pX_{2} \right]$ | (34) |
| --- | --- |

By changing the total protein abundances, MRA-based reconstruction was performed for a large range of the weight parameter $a$ values. For either selection of module outputs (Supplementary Equations 32‑34), at low $a$ values, both connection coefficients, $r_{21}$ and $r_{12}$, were negative (Supplementary Figures 1B-1D, point 1). For the choice of communicating species shown in Supplementary Equation 32, only the sequestration-induced connection $r_{21}$ changed its sign with the increase in weight $a$ (point 2, Supplementary Figure 1B). For the choice of communicating species as in Supplementary Equation 33, only the regulatory connection $r_{12}$ changed its sign with the increase in weight $a$ (point 2, Supplementary Figure 1C). In case when the protein-protein complex, $pX_{1}\text{∙∙}pX_{2}$, (weighted by $a$) was added to the outputs of both modules (Supplementary Equation 34), both connections, $r_{21}$ and $r_{12}$, changed their signs (points 2 and 3 in Supplementary Figure 1D). Importantly, for either selection of module outputs (Supplementary Equations 32-34), the connections changed their signs at the same values of $a$ (compare points 2 in Supplementary Figures 1B-1C with points 2 and 3 in Supplementary Figures 1D). Analytical investigation showed that only at point 2 on Supplementary Figure 1B and point 2 on Supplementary Figure 1D the modular insulation condition is restored (see attached Sage worksheet “inh_cascade.sws”). At point 2, for the communicating species defined by Supplementary Equation 32, the connection matrix has the following form

| $r_{ij}=\left( \begin{matrix} -1 & 0 \\ -0.26 & -1 \end{matrix} \right)$ | (35). |
| --- | --- |

The permissible parameters are: 1) $X_{1}^{tot}$ for module 1 and 2) $X_{1}^{tot}$, $k_{31}$, $k_{32}$, $k_{4}$, $k_{5}$ and $k_{6}$ for module 2.

For communicating species defined by Supplementary Equation 34, the connection matrix is the following at point 2,

| $r_{ij}=\left( \begin{matrix} -1 & 0 \\ -0.2 & -1 \end{matrix} \right)$ | (36). |
| --- | --- |

The permissible parameters are the same as in Supplementary Equation 35.

Thus, in case of cascades where an upstream module inhibits, rather than activates, the downstream module (as shown in Supplementary Figure 1A) and appreciable sequestration effects, our approach allows for the distinguishing between sequestration and regulatory connections. However, it delivers a unique connection coefficient matrix only if the information of enzyme-substrate relations is available. For example, if co-immunoprecipitation detects a significant complex formation between proteins from different modules, additional information such as consensus phosphorylation sequences for many kinases can be utilized to detect sequestration-induced connections and infer unique connection matrix invariant to the set of perturbations used. Communicating species can be defined as in Supplementary Equation 32. In this case, only the sequestration connections change their signs for a certain optimal set of weight parameters $\vec{a}=\vec{a}^{opt}$.

**A B**


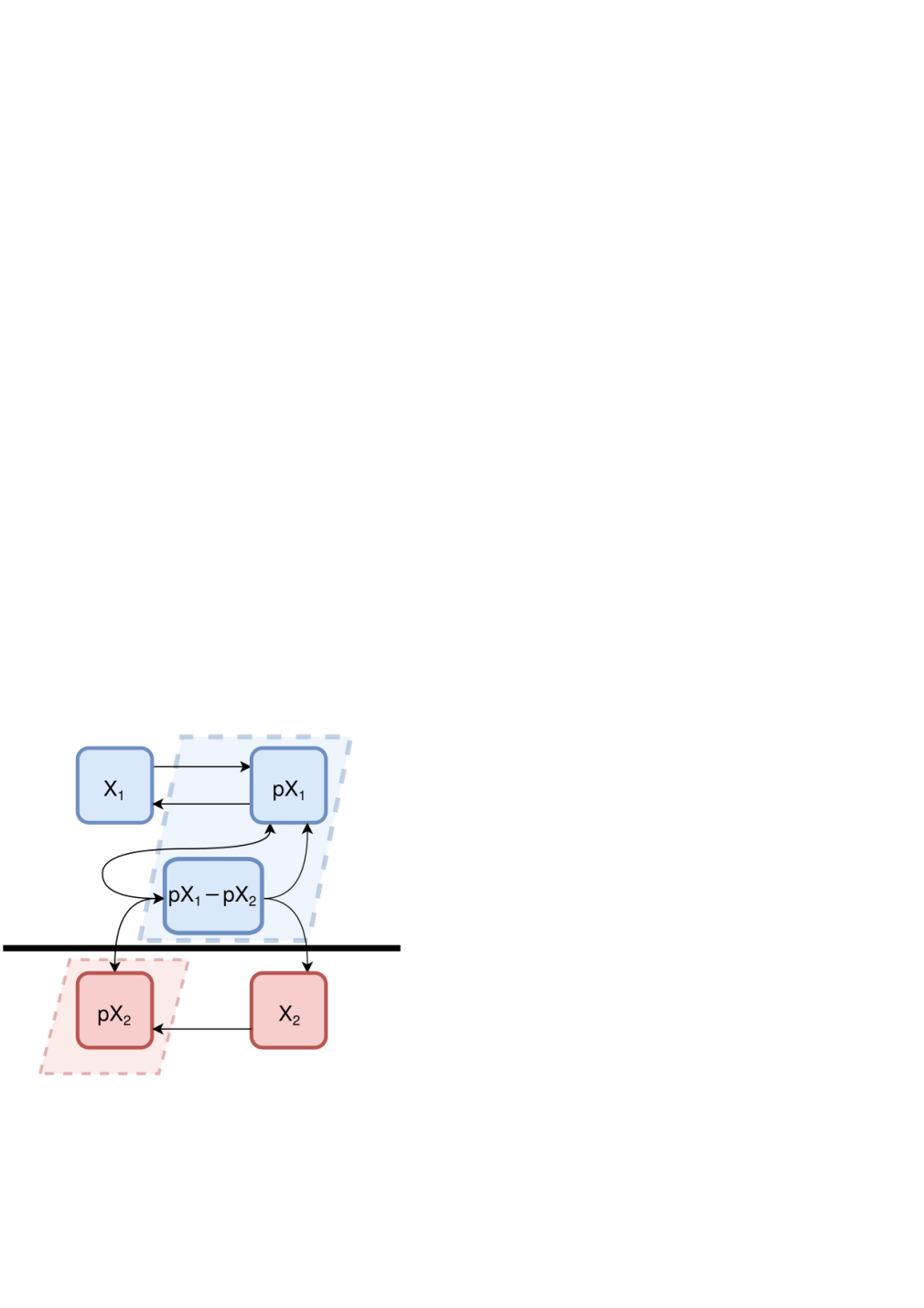

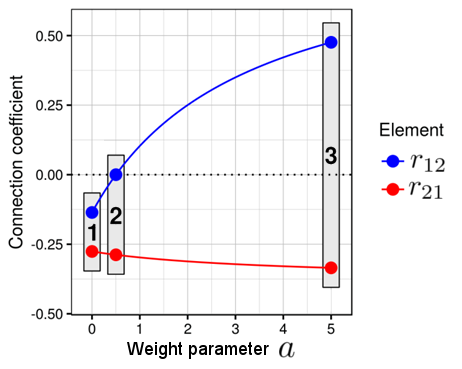


**C D**


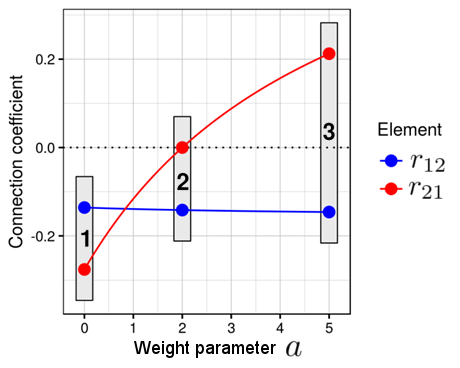

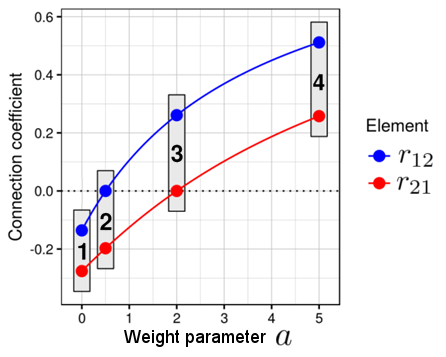


**Supplementary Figure 1. Dependence of the elements of the connection matrix for a two-tier inhibitory cascade model on the weight parameter a. (A).** Reaction scheme of two cascade modules. **(B).** Only the sequestration connection ($r_{12}$, shown in blue) changes the sign with the increase in a weight parameter ($a)$ when the protein-protein complex concentration is added to the first module output (Supplementary Equation 32). **(B).** Only regulatory connection ($r_{21}$, shown in red) changes the sign with the increase in a weight parameter ($a$) when the protein-protein complex concentration is added to the second module output (Supplementary Equation 33). **(D)**. Both regulatory and sequestration connections change their sign with the increase in $a$ (points 2 and 3) when the protein-protein complex (weighted by $a$) is added to both module outputs (Supplementary Equation 34).

| **Supplementary Table 12. Kinetic equations of the model of a two-tier inhibitory cascade** | |
| --- | --- |
| $d\left[ X_{1} \right]/dt$ | $-k_{1}\left[ X_{1} \right]+k_{2}\left[ pX_{1} \right]$ |
| $d\left[ pX_{1} \right]/dt$ | $k_{1}\left[ X_{1} \right]-k_{2}\left[ pX_{1} \right]-k_{31}\left[ pX_{1} \right]\left[ pX_{2} \right]+\left( k_{32}+k_{4} \right)\left[ pX_{1}\text{∙∙}pX_{2} \right]$ |
| $d\left[ pX_{1}\text{∙∙}pX_{2} \right]/dt$ | $k_{31}\left[ pX_{1} \right]\left[ pX_{2} \right]-\left( k_{32}+k_{4} \right)\left[ pX_{1}\text{∙∙}pX_{2} \right]$ |
| $d\left[ X_{2} \right]/dt$ | $k_{31}\left[ pX_{1} \right]\left[ pX_{2} \right]-\left( k_{32}+k_{4} \right)\left[ pX_{1}\text{∙∙}pX_{2} \right]$ |
| $d\left[ pX_{2} \right]/dt$ | $k_{5}\left[ X_{2} \right]-k_{6}\left[ pX_{2} \right]-k_{31}\left[ pX_{1} \right]\left[ pX_{2} \right]+k_{32}\left[ pX_{1}\text{∙∙}pX_{2} \right]$ |
| Moiety conservation relations | $X_{1}^{tot}=\left[ X_{1} \right]+\left[ pX_{1} \right]+\left[ pX_{1}\text{∙∙}pX_{2} \right]$  $X_{2}^{tot}=\left[ X_{2} \right]+\left[ pX_{2} \right]+\left[ pX_{1}\text{∙∙}pX_{2} \right]$ |

| **Supplementary Table 13. Parameter values of the model of a two-tier inhibitory cascade with feedback and sequestration connections between the same modules** | |
| --- | --- |
| $X_{1}^{tot}$ | 100 nM |
| $X_{2}^{tot}$ | 100 nM |
| $k_{1}$ | 0.1 1/s |
| $k_{2}$ | 0.1 1/s |
| $k_{31}$ | 0.01 nM^-1^s^-1^ |
| $k_{32}$ | 1 s^-1^ |
| $k_{4}$ | 0.1 s^-1^ |
| $k_{5}$ | 0.1 s^-1^ |
| $k_{6}$ | 0.5 s^-1^ |

# Section 7. Model of a signaling hub

When a protein, e.g. a kinase phosphorylates and regulates more than one protein, a hub-like signaling motif is observed (Supplementary Figure 2). The model equations are presented in Supplementary Table 14 and parameter values are given in Supplementary Table 15. For this hub-like motif, sequestration-induced, retroactive connections occur not only between the upstream module X and downstream modules Y and Z, but this retroactivity also influences the connections between Y and Z. This leads to four elements of the connection matrix which exist only due to sequestration.

We will define the communicating species as follows:

| $\begin{matrix} x_{1}^{a}= \left[ pX \right]+a_{[pX\text{∙∙}Y]}\cdot\left[ pX\text{∙∙}Y \right]+a_{\left[ pX\text{∙∙}pZ \right]}\cdot\left[ pX\text{∙∙}pZ \right] \\ x_{2}^{a}=[pY] \\ x_{3}^{a}=[pZ] \end{matrix}$ | (37) |
| --- | --- |
|  |  |
| $\begin{matrix} x_{1}^{a}= \left[ pX \right]+a_{[pX\text{∙∙}Y]}\cdot\left[ pX\text{∙∙}Y \right]+a_{\left[ pX\text{∙∙}pZ \right]}\cdot\left[ pX\text{∙∙}pZ \right] \\ x_{2}^{a}=[pY]+a_{[pX\text{∙∙}Y]}\cdot\left[ pX\text{∙∙}Y \right] \\ x_{3}^{a}=[pZ]+a_{\left[ pX\text{∙∙}pZ \right]}\cdot\left[ pX\text{∙∙}pZ \right] \end{matrix}$ | (38) |

For both definitions Supplementary Equations 37 and 38, the elements of the local response matrix representing connections, induced by retroactivity of Y and X, as well as by retroactivity of Z and X, change their sign at *different* sets of the weight parameters $a_{[pX\text{∙∙}Y]}$ and $a_{\left[ pX\text{∙∙}pZ \right]}$. Thus, it is impossible to find a single weight parameter set, which satisfies modular insulation condition (Eqs. 6 and 11).

Nevertheless, defining the communicating species by Supplementary Equations 37 or 38 and scanning through the values of the weight parameters, correctly identifies the retroactivity connections (induced by sequestration). For example, calculating the connection matrices by perturbing the total abundances, $X^{tot}$, $Y^{tot}$ and $Z^{tot}$, with the given parameter set and communicating species that are dependent on $a_{i}$, as given by Supplementary Equation 37, yields,

| $a_{\left[ pX\text{∙∙}Y \right]}=a_{\left[ pX\text{∙∙}pZ \right]}=0$:  $r_{ij}=\left( \begin{matrix} -1 & -0.44 & -0.44 \\ 0.55 & -1 & 0.02 \\ 0.16 & 0.01 & -1 \end{matrix} \right)$ | $a_{\left[ pX\text{∙∙}Y \right]}=a_{\left[ pX\text{∙∙}pZ \right]}=1$:  $r_{ij}=\left( \begin{matrix} -1 & 0.08 & 0.09 \\ 1.33 & -1 & -0.33 \\ 0.47 & -0.09 & -1 \end{matrix} \right)$. | (39) |
| --- | --- | --- |

Here, the connection coefficients marked red change their sign with changing the weights ($a_{[pX\text{∙∙}Y]}$ and $a_{\left[ pX\text{∙∙}pZ \right]}$) and arise solely from protein sequestration. Although this shows that the method correctly identifies the sequestration connections, they cannot be nullified at a single weight parameter set. The minimization of the sum of the squares of sequestration connection coefficients at some $\vec{a}=\vec{a}^{min}$ gives the matrix, which correctly infers the activating regulatory connections and shows the smallest absolute values for other connections that are related to Y to X and Z to X retroactivity.

| $a_{\left[ pX\text{∙∙}Y \right]}=a_{\left[ pX\text{∙∙}Y \right]}^{min}=0.65, a_{\left[ pX\text{∙∙}pZ \right]}=a_{\left[ pX\text{∙∙}pZ \right]}^{min}=0.39$  $r_{ij}=\left( \begin{matrix} -1 & 0.01 & -0.10 \\ 0.95 & -1 & -0.13 \\ 0.33 & -0.06 & -1 \end{matrix} \right)$. | (40) |
| --- | --- |

Interestingly, the minimization of the sum of the squares of sequestration connection coefficients also minimizes the difference between the connection matrices $r_{ij}$ that are inferred, using alternative perturbations. For example, the connection matrices obtained by perturbing parameters $v_{1}$, $k_{31}$ and $Z^{tot}$ are the following

| $a_{\left[ pX\text{∙∙}Y \right]}=a_{\left[ pX\text{∙∙}pZ \right]}=0$:  $r_{ij}=\left( \begin{matrix} -1.00 & -0.44 & -0.55 \\ 0.52 & -1.00 & 0.01 \\ 0.15 & 0.00 & -1.00 \end{matrix} \right)$ | $a_{\left[ pX\text{∙∙}Y \right]}=a_{\left[ pX\text{∙∙}pZ \right]}=1$:  $r_{ij}=\left( \begin{matrix} -1.00 & 0.08 & 0.10 \\ 1.27 & -1.00 & -0.32 \\ 0.44 & -0.09 & -1.00 \end{matrix} \right)$. | (41) |
| --- | --- | --- |

| $a_{\left[ pX\text{∙∙}Y \right]}=a_{\left[ pX\text{∙∙}Y \right]}^{min}=0.7, a_{\left[ pX\text{∙∙}pZ \right]}=a_{\left[ pX\text{∙∙}pZ \right]}^{min}=0.3$  $r_{ij}=\left( \begin{matrix} -1.00 & 0.01 & -0.10 \\ 0.91 & -1.00 & -0.13 \\ 0.31 & -0.07 & -1.00 \end{matrix} \right)$. | (42) |
| --- | --- |

Thus, although in this case our heuristic approach cannot obtain the connection matrix, which would be invariant to different perturbations, it minimizes the discrepancy between the inferred matrices (the difference between connection coefficients and the L_2_ matrix norms is much greater in Supplementary Equations 39 and 41, than this difference in Supplementary Equations 40 and 42). Importantly, sequestration connections are most variable elements of a connection matrix obtained using different perturbation sets (see Supplementary Equation 39 and 41). Therefore, it is not surprising that the minimization of sequestration connections decreases discrepancies between the matrices inferred from different perturbation sets (see Supplementary Equation 40 and 42).


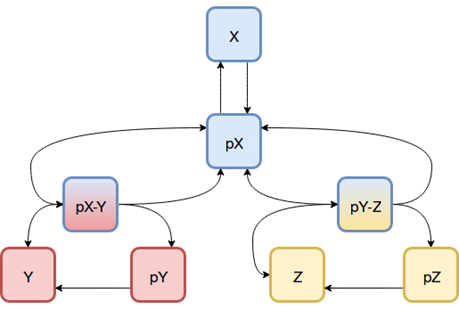


**Supplementary Figure 2. Reaction scheme of the model of a signaling hub.**

| **Supplementary Table 14. Kinetic equations of the model of enzymatic signaling hub** | |
| --- | --- |
| $d\left[ X \right]/dt$ | $-v_{1}\left[ X \right]/\left( k_{1}+\left[ X \right] \right)+v_{2}\left[ pX \right]/\left( k_{2}+\left[ pX \right] \right)$ |
| $d\left[ pX \right]/dt$ | $v_{1}\left[ X \right]/\left( k_{1}+\left[ X \right] \right)-v_{2}\left[ pX \right]/\left( k_{2}+\left[ pX \right] \right)-k_{31}\left[ Y \right]\left[ pX \right]+k_{32}\left[ pX\text{∙∙}Y \right]+k_{4}\left[ pX\text{∙∙}Y \right]-k_{61}\left[ Z \right]\left[ pX \right]+k_{62}\left[ pX\text{∙∙}Z \right]+k_{7}\left[ pX\text{∙∙}Z \right]$ |
| $d\left[ pX\text{∙∙}Y \right]/dt$ | $k_{31}\left[ Y \right]\left[ pX \right]-k_{32}\left[ pX\text{∙∙}Y \right]-k_{4}\left[ pX\text{∙∙}Y \right]$ |
| $d\left[ Y \right]/dt$ | $-k_{31}\left[ Y \right]\left[ pX \right]+k_{32}\left[ pX\text{∙∙}Y \right]+v_{5}\left[ pY \right]/\left( k_{5}+\left[ pY \right] \right)$ |
| $d\left[ pY \right]/dt$ | $k_{4}\left[ pX\text{∙∙}Y \right]-v_{5}\left[ pY \right]/\left( k_{5}+\left[ pY \right] \right)$ |
| $d\left[ pX\text{∙∙}Z \right]/dt$ | $k_{61}\left[ Z \right]\left[ pX \right]-k_{62}\left[ pX\text{∙∙}Z \right]-k_{7}\left[ pX\text{∙∙}Z \right]$ |
| $d\left[ Z \right]/dt$ | $-k_{61}\left[ Z \right]\left[ pX \right]+k_{62}\left[ pX\text{∙∙}Z \right]+v_{8}\left[ pZ \right]/\left( k_{8}+\left[ pZ \right] \right)$ |
| $d\left[ pZ \right]/dt$ | $k_{7}\left[ pX\text{∙∙}Z \right]-v_{8}\left[ pZ \right]/\left( k_{8}+\left[ pZ \right] \right)$ |

| **Supplementary Table 15. Parameter values for the model of enzymatic signaling hub** | |
| --- | --- |
| $X^{tot}$ | 100 nM |
| $Y^{tot}$ | 100 nM |
| $Z^{tot}$ | 100 nM |
| $k_{1}$ | 100 nM |
| $v_{1}$ | 1.5 nM/s |
| $k_{2}$ | 100 nM |
| $v_{2}$ | 1 nM/s |
| $k_{31}$ | 0.01 nM^-1^s^-1^ |
| $k_{32}$ | 0.4 s^-1^ |
| $k_{4}$ | 0.05 s^-1^ |
| $k_{5}$ | 100 nM |
| $v_{5}$ | 5 nM/s |
| $k_{61}$ | 0.05 nM^-1^s^-1^ |
| $k_{62}$ | 0.4 s^-1^ |
| $k_{7}$ | 0.05 s^-1^ |
| $k_{8}$ | 100 nM |
| $v_{8}$ | 5 nM/s |

# Section 8. Model combining the hub and cascade signaling motifs

We showed that our approach allows to unmistakably infer network connections for activation signaling cascades that have waterfall circuitry, similar to the topology of MAPK cascades, including feedbacks. However, for signaling hub circuities, we cannot infer connection matrices that are invariant to a wide range of experimental perturbations. Therefore, we next test this approach performs for a network that combines a hub and waterfall network motifs. A simplified scheme of this network is given in Supplementary Figure 3. All regulatory influences are modeled mechanistically, using mass-action kinetics. The corresponding equations and parameters are given in Supplementary Table 16 and Supplementary Table 17.

Similarly to a hub case, a unique set $\vec{a}=\vec{a}^{opt}$ that nullifies all sequestration-induced connections at once cannot be found. Therefore, we vary weight parameters $\vec{a}$ to minimize the sum of squares of sequestration connections, resulting in a parameter set $\vec{a}=\vec{a}^{min}$. Supplementary Equation 43-45 demonstrate that at $\vec{a}=\vec{a}^{min}$, sequestration feedback connections from module T to any other upstream module become zero. Module T is more distant from the hub X than modules Y, Z and W. In fact, sequestration connections between modules Y and Z and between modules W and Y are not nullified, but the sequestration feedback connections to module U are also zero at $\vec{a}=\vec{a}^{min}$. Thus, our approach allows for a complete elimination of solely retroactivity effects upstream and downstream of signaling hubs, although the sequestration connections in the vicinity of a signaling hub cannot be simultaneously nullified.


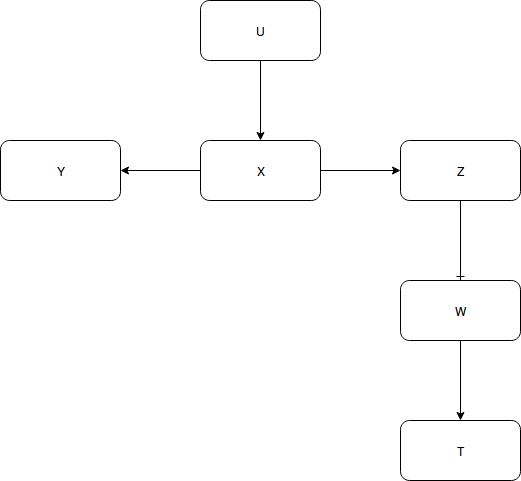


Supplementary Figure 3. A simplified scheme of a model that combines cascade and hub signaling motifs

Supplementary Table 16. Kinetic equations of the model that combines cascade and hub signaling motifs

| $d\left[ u_{1} \right]/dt$ | $-v_{1}\left[ u_{1} \right]/(K_{m1}+\left[ u_{1} \right]) +v_{2}\left[ u_{2} \right]/(K_{m2}+\left[ u_{2} \right])$ |
| --- | --- |
| $d\left[ u_{2} \right]/dt$ | $v_{1}\left[ u_{1} \right]/(K_{m1}+\left[ u_{1} \right])-v_{2}\left[ u_{2} \right]/(K_{m2}+\left[ u_{2} \right])-k_{91}\left[ u_{2} \right]\left[ x_{1} \right]+k_{92}\left[ u_{2}\text{∙∙}x_{1} \right]+k_{9}^{cat}\left[ u_{2}\text{∙∙}x_{1} \right]$ |
| $d\left[ x_{1} \right]/dt$ | $-k_{91}\left[ u_{2} \right]\left[ x_{1} \right]+k_{92}\left[ u_{2}\text{∙∙}x_{1} \right]+v_{10}\left[ x_{2} \right]/(K_{m10}+\left[ x_{2} \right])$ |
| $d\left[ u_{2}\text{∙∙}x_{1} \right]/dt$ | $k_{91}\left[ u_{2} \right]\left[ x_{1} \right]-k_{92}\left[ u_{2}\text{∙∙}x_{1} \right]-k_{9}^{cat}\left[ u_{2}\text{∙∙}x_{1} \right]$ |
| $d\left[ x_{2} \right]/dt$ | $k_{9}^{cat}\left[ u_{2}\text{∙∙}x_{1} \right]-v_{10}\left[ x_{2} \right]/\left( K_{m10}+\left[ x_{2} \right] \right)-k_{31}\left[ y_{1} \right]\left[ x_{2} \right]+k_{32}\left[ x_{2}\text{∙∙}y_{1} \right]+k_{3}^{cat}\left[ x_{2}\text{∙∙}y_{1} \right]\left( 1+k_{cz}\left[ z_{2} \right] \right)/\left( 1+\left[ z_{2} \right] \right)-k_{61}\left[ z_{1} \right]\left[ x_{2} \right]+k_{62}\left[ x_{2}\text{∙∙}z_{1} \right]+k_{6}^{cat}\left[ x_{2}\text{∙∙}z_{1} \right]$ |
| $d\left[ y_{1} \right]/dt$ | $-k_{31}\left[ y_{1} \right]\left[ x_{2} \right]+k_{32}\left[ x_{2}\text{∙∙}y_{1} \right]+v_{5}\left[ y_{2} \right]/(K_{m5}+\left[ y_{2} \right])$ |
| $d\left[ x_{2}\text{∙∙}y_{1} \right]/dt$ | $k_{31}\left[ y_{1} \right]\left[ x_{2} \right]-k_{32}\left[ x_{2}\text{∙∙}y_{1} \right]-k_{3}^{cat}\left[ x_{2}\text{∙∙}y_{1} \right](1+k_{cz}\left[ z_{2} \right])/(1+\left[ z_{2} \right])$ |
| $d\left[ y_{2} \right]/dt$ | $k_{3}^{cat}\left[ x_{2}\text{∙∙}y_{1} \right]\left( 1+k_{cz}\left[ z_{2} \right] \right)/(1+\left[ z_{2} \right])-v_{5}\left[ y_{2} \right]/(K_{m5}+\left[ y_{2} \right])$ |
| $d\left[ z_{1} \right]/dt$ | $-k_{61}\left[ z_{1} \right]\left[ x_{2} \right]+k_{62}\left[ x_{2}\text{∙∙}z_{1} \right]+v_{8}\left[ z_{2} \right]/(K_{m8}+\left[ z_{2} \right])$ |
| $d\left[ x_{2}\text{∙∙}z_{1} \right]/dt$ | $k_{61}\left[ z_{1} \right]\left[ x_{2} \right]-k_{62}\left[ x_{2}\text{∙∙}z_{1} \right]-k_{6}^{cat}\left[ x_{2}\text{∙∙}z_{1} \right]$ |
| $d\left[ z_{2} \right]/dt$ | $k_{6}^{cat}\left[ x_{2}\text{∙∙}z_{1} \right]-v_{8}\left[ z_{2} \right]/(K_{m8}+\left[ z_{2} \right])-k_{111}\left[ z_{2} \right]\left[ w_{2} \right]+k_{112}\left[ z_{2}\text{∙∙}w_{2} \right]+k_{11}^{cat}\left[ z_{2}\text{∙∙}w_{2} \right]$ |
| $d\left[ w_{2} \right]/dt$ | $-k_{111}\left[ z_{2} \right]\left[ w_{2} \right]+k_{112}\left[ z_{2}\text{∙∙}w_{2} \right]+v_{12}\left[ w_{1} \right]/(K_{m12}+\left[ w_{1} \right])-k_{131}\left[ w_{2} \right]\left[ s_{1} \right]+k_{132}\left[ w_{2}\text{∙∙}s_{1} \right]+k_{13}^{cat}\left[ w_{2}\text{∙∙}s_{1} \right]$ |
| $d\left[ z_{2}\text{∙∙}w_{2} \right]/dt$ | $k_{111}\left[ z_{2} \right]\left[ w_{2} \right]-k_{112}\left[ z_{2}\text{∙∙}w_{2} \right]-k_{11}^{cat}\left[ z_{2}\text{∙∙}w_{2} \right]$ |
| $d\left[ w_{1} \right]/dt$ | $k_{11}^{cat}\left[ z_{2}\text{∙∙}w_{2} \right]-v_{12}\left[ w_{1} \right]/(K_{m12}+\left[ w_{1} \right])$ |
| $d\left[ s_{1} \right]/dt$ | $-k_{131}\left[ w_{2} \right]\left[ s_{1} \right]+k_{132}\left[ w_{2}\text{∙∙}s_{1} \right]+v_{14}\left[ s_{2} \right]/(K_{m14}+\left[ s_{2} \right])$ |
| $d\left[ w_{2}\text{∙∙}s_{1} \right]/dt$ | $k_{131}\left[ w_{2} \right]\left[ s_{1} \right]-k_{132}\left[ w_{2}\text{∙∙}s_{1} \right]-k_{13}^{cat}\left[ w_{2}\text{∙∙}s_{1} \right]$ |
| $d\left[ s_{2} \right]/dt$ | $k_{13}^{cat}\left[ w_{2}\text{∙∙}s_{1} \right]-v_{14}\left[ s_{2} \right]/(K_{m14}+\left[ s_{2} \right])$ |

Supplementary Table 17. Parameter values for the model that combines cascade and hub signaling motifs

| $U^{tot}$ | 100 nM |
| --- | --- |
| $X^{tot}$ | 100 nM |
| $Y^{tot}$ | 100 nM |
| $Z^{tot}$ | 100 nM |
| $W^{tot}$ | 100 nM |
| $S^{tot}$ | 100 nM |
| $K_{m1}$ | 100.00 nM |
| $v_{1}$ | 1.00 nM/s |
| $K_{m2}$ | 100.00 nM |
| $v_{2}$ | 1.00 nM/s |
| $k_{31}$ | 0.01 nM^-1^s^-1^ |
| $k_{32}$ | 0.40 s^-1^ |
| $k_{3}^{cat}$ | 0.05 s^-1^ |
| $K_{m5}$ | 100.00 nM |
| $v_{5}$ | 5.00 nM/s |
| $k_{61}$ | 0.05 nM^-1^s^-1^ |
| $k_{62}$ | 0.40 s^-1^ |
| $k_{6}^{cat}$ | 0.01 s^-1^ |
| $K_{m8}$ | 100.00 nM |
| $v_{8}$ | 1.00 nM/s |
| $k_{91}$ | 0.01 nM^-1^s^-1^ |
| $k_{92}$ | 0.50 s^-1^ |
| $k_{9}^{cat}$ | 0.01 s^-1^ |
| $K_{m10}$ | 100.00 nM |
| $v_{10}$ | 1.50 nM/s |
| $k_{111}$ | 0.01 nM^-1^s^-1^ |
| $k_{112}$ | 0.40 s^-1^ |
| $k_{11}^{cat}$ | 0.05 s^-1^ |
| $K_{m12}$ | 100.00 nM |
| $v_{12}$ | 1.50 nM/s |
| $k_{131}$ | 0.01 nM^-1^s^-1^ |
| $k_{132}$ | 0.40 s^-1^ |
| $k_{13}^{cat}$ | 0.05 s^-1^ |
| $K_{m14}$ | 100.00 nM |
| $v_{14}$ | 1.50 nM/s |
| $k_{cz}$ | 1.00 |

| $\left. r_{ij} \right\vert_{a_{k}=0 \forall k}=\left( \begin{matrix} -1.00 & -0.17 & 0 & 0.01 & 0 & 0 \\ 0.46 & -1.00 & -0.30 & -0.49 & 0 & 0 \\ 0 & 0.73 & -1.00 & 0.01 & 0 & 0 \\ 0 & 0.26 & 0 & -1.00 & -0.13 & 0 \\ 0 & 0 & 0 & -0.88 & -1.00 & -0.05 \\ 0 & 0 & 0 & 0.03 & 0.41 & -1.00 \end{matrix} \right)$ | (43) |
| --- | --- |

| $\left. r_{ij} \right\vert_{a_{k}=0 \forall k}=\left( \begin{matrix} -1.00 & 0.60 & -0.14 & -0.23 & 0.10 & -0.03 \\ 0.12 & -1.00 & 0.17 & 0.29 & -0.12 & 0.03 \\ -0.01 & 1.99 & -1.00 & -0.74 & 0.31 & -0.08 \\ -0.00 & 0.85 & -0.19 & -1.00 & 0.27 & -0.07 \\ 0.00 & 0.01 & -0.00 & -0.67 & -1.00 & 0.23 \\ -0.00 & -0.01 & 0.00 & 0.04 & 0.58 & -1.00 \end{matrix} \right)$ | (44) |
| --- | --- |

| $\left. r_{ij} \right\vert_{a_{k}=a_{k}^{min}}=\left( \begin{matrix} -1.00 & 0.01 & -0.00 & -0.00 & 0.00 & -0.00 \\ 0.30 & -1.00 & 0.01 & -0.12 & 0.02 & -0.00 \\ -0.01 & 1.04 & -1.00 & -0.14 & 0.02 & -0.00 \\ -0.00 & 0.41 & -0.07 & -1.00 & -0.00 & 0.00 \\ -0.00 & -0.00 & 0.00 & -0.91 & -1.00 & -0.00 \\ -0.00 & -0.00 & 0.00 & 0.03 & 0.43 & -1.00 \end{matrix} \right)$ | (45) |
| --- | --- |

# Section 9 Application of the proposed method using noisy experimental data

Noisy experimental data are a challenge for all network reconstruction techniques. In the case of MRA, statistical reformulations have been proposed to facilitate the determination of local response coefficients from noisy data [^4^](#_ENREF_4). In our recent publication [^5^](#_ENREF_5), impact of measurement noise and perturbation magnitudes on the original MRA procedure are assessed. We concluded that, although local response coefficients follow a heavy-tailed distribution for high levels of noise, MRA can safely be applied for low levels of experimental noise.

In order to investigate impact of noisy data on our proposed method, we sampled data for the model of the three tier cascade (with no feedback connections) presented in Supplementary material Section 4 (Supplementary Tables 2 and 3). The simulations are also available as a reproducible RMarkdown script. The response data were simulated with the model parameters presented in Supplementary material Section 4. Each data point was simulated according to

| $x=x_{SS}(p)+\epsilon,$ | (46) |
| --- | --- |

where $x_{SS}\left( p \right)$ refers to the steady state solution of the ODEs of Supplementary Table 2 with the corresponding parameter set of Supplementary Table 3, and $\epsilon\sim N\left( 0,\sigma^{2} \right)$ with relative error model $\sigma=s_{rel}*x_{SS}$. Various noise levels were tested by tuning the error parameter, $s_{rel}$. We simulated with noise levels 0.1%, 1%, 5% and 10%. For the perturbations of modules, parameter perturbations of total concentrations were simulated by decreasing the total concentrations by 50% (Supplementary Figure 4). For each setting, 1000 data realizations (data samples) were simulated. The distributions of the local response coefficients were calculated using the MRA equations for two different module outputs, in one case they were selected as in standard MRA (the weight parameter $\vec{a}=0$) and in the other case inter-modular enzyme-substrate complexes were added to active free forms of signaling enzymes with the weight parameter $\vec{a}=\vec{a}^{opt}$. The weight parameter $\vec{a}^{opt}$ was calculated separately for each data sample.

The results for parameter perturbations with the 50% magnitude can be seen in Supplementary Figures 4 and 5. Supplementary Figure 4 presents distributions of connection coefficients values, inferred for varying noise levels and for the standard and optimized selection of communicating species. In panel D of Supplementary Figure 4, the y-axes were cut to the range between [-2, 2] to enhance the visibility of the quartiles of the distributions. Supplementary Figure 5 presents statistics of how often each connection was correctly identified as a sequestration connection or a regulatory connection during data sampling. For both figures, panels A-D correspond to different noise levels with standard deviations 0.1%, 1%, 5% and 10% of the absolute value of the steady state concentrations. Each panel is organized in subpanels, which visually represent the distributions of the connection matrix coefficients. Within each subpanel, two box plots are shown for the distributions of the local response matrix elements for two different choices of the weight parameters $\vec{a}$. The distributions for $\vec{a}=0$ are shown on the left, and the distribution for $\vec{a}=\vec{a}^{opt}$ are shown on the right.

As observed in our earlier publication[^5^](#_ENREF_5), the distributions of the local response coefficients are heavy-tailed and their width increases with increasing noise (Supplementary Figure 4). For low noise levels, both sequestration and regulatory connections are identified correctly (Supplementary Figure 5). Optimization of the weight parameters $\vec{a}$, clearly shows (see Supplementary Figure 4) that sequestration influences the connection coefficients to a larger degree than noise. Moreover, the accuracy of the local response matrix inference profits from the optimal choice of communicating species (the weight parameter $\vec{a}=\vec{a}^{opt})$. For high noise levels, the distributions become broader and retroactivity is not the dominating effect anymore (Supplementary Figure 4). Yet, the type of connections (regulatory or sequestration) is correctly identified in most cases (Supplementary Figure 5, note that incorrect identification of connection coefficient $r_{31}$ happens only because of a very small absolute value of this coefficient, which is much lower than other connections in the matrix, see also Supplementary Figure 4) and the medians of sequestration-induced connection coefficients correctly shift to zero even for high levels of noise (Supplementary Figure 4). However, for noisy data deterministic MRA should generally be used with a great caution.

A way to improve the performance of network inference is to average the data from some number of replicates, as we recently have shown[^5^](#_ENREF_5). Experimental data replicates allow for the estimation of the noise magnitude. Given such information on the standard deviation of noisy experimental data, a sampling procedure can be applied to the average of data replicates, using the error model given by Supplementary Equation 46 and calculating the connection coefficients in the exactly same way as it is done in this section. To estimate the confidence intervals for connection coefficients and their type, the computational noise level should be equal to noise level estimated from experiments. Similar procedure was successfully applied previously to estimate confidence intervals for the connection coefficients[^6^](#_ENREF_6). However, the robust improvement of network inference with respect to noise requires to resort to statistical MRA reformulations, which account for uncertainty, such as the Bayesian variant of MRA (BMRA)[^7^](#_ENREF_7). Since BMRA uses MRA equations (Eq. 7) to estimate posterior likelihoods, incorporation of our approach into BMRA is straightforward.

| A  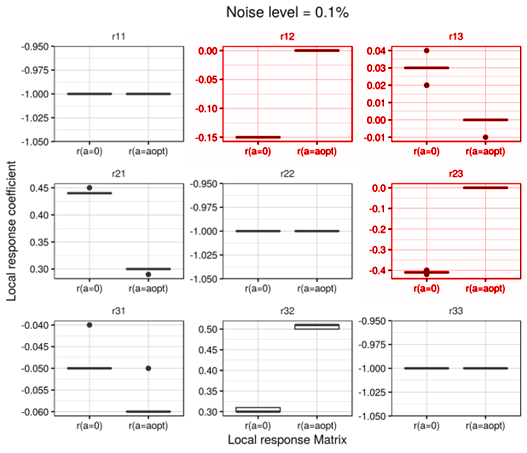 | B  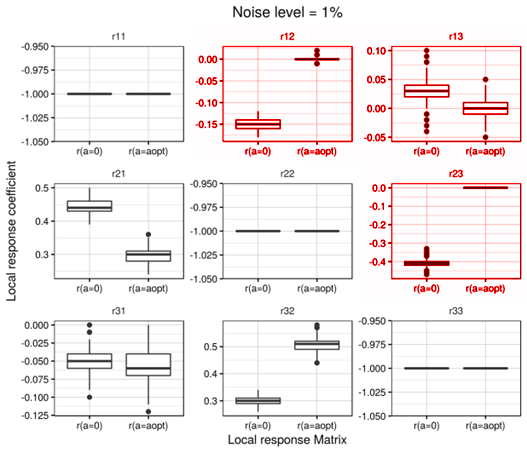 |
| --- | --- |
| C  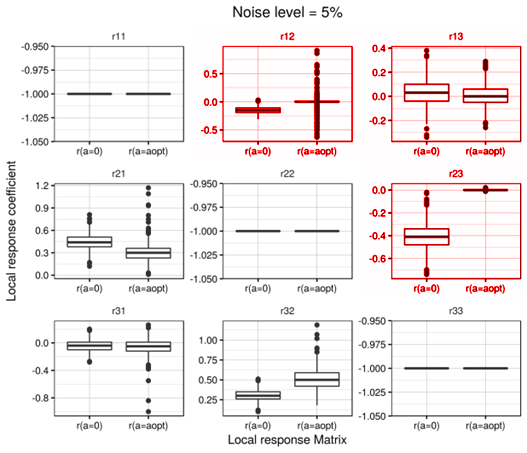 | D  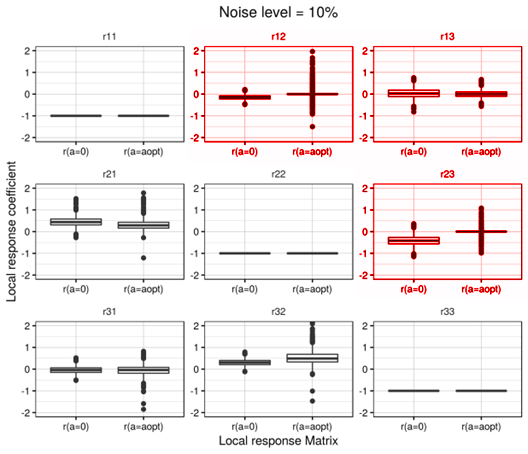 |

**Supplementary Figure 4: Distributions of connection coefficients inferred for varying noise levels and for the standard and optimized selection of communicating species.** Experimental perturbations of modules were simulated by decreasing the corresponding total abundances by 50%. Panels a-d refer to different, indicated noise levels. Each panel is organized in subpanels, which correspond to the connection coefficients in the same positions as they have in the connection matrix (e.g, in panel a in the 2^nd^ row, the 1^st^ column displays the distributions for the regulatory connection coefficient, $r_{21}$). For each connection coefficient, the distributions for two different choices of the vector of weight parameters $\vec{a}$ are shown. On the left hand side of each subpanel, the weight parameters  $\vec{a}$ are set to zero (standard selection of communicating species), whereas on the right hand side, the optimal weight parameters $\vec{a}=\vec{a}^{opt}$ are chosen (optimized selection of communicating species). Coefficients, $r_{21}$, $r_{31}$ and $r_{32}$ (black color) are regulatory connection coefficients, and coefficients $r_{13}$, $r_{12}$ and $r_{23}$ (red color) are sequestration connection coefficients (see Supplementary material Section 4).

| A  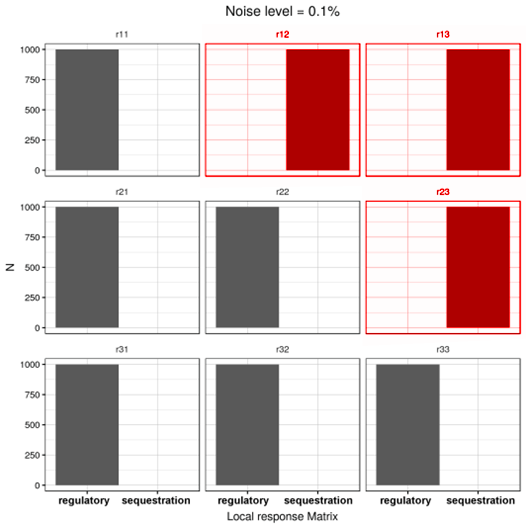 | B  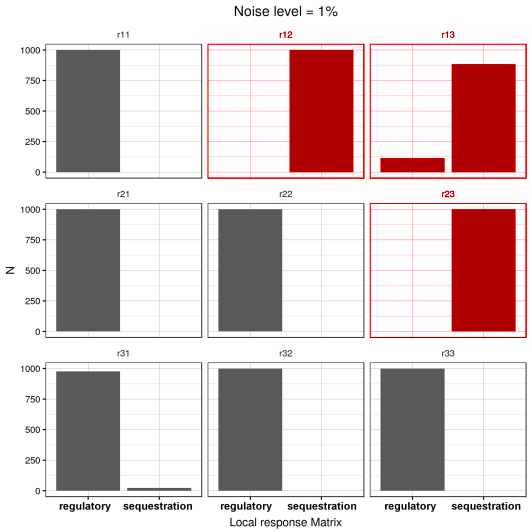 |
| --- | --- |
| C  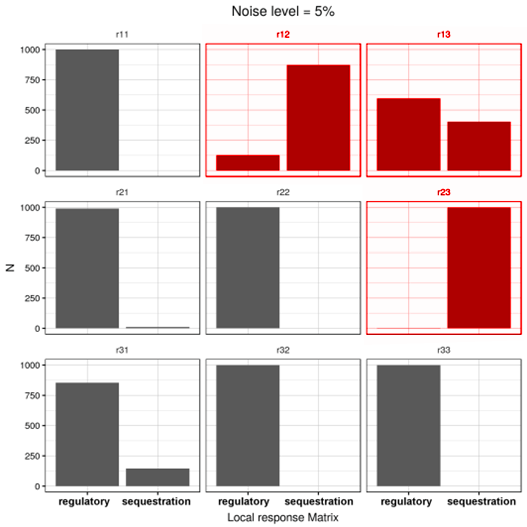 | D  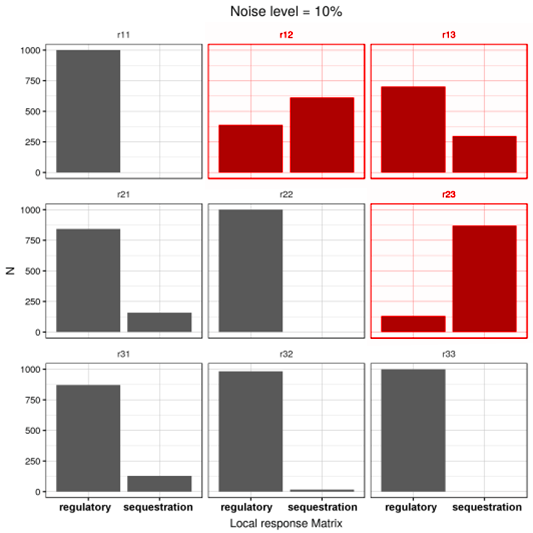 |

**Supplementary Figure 5: Frequencies of correct vs incorrect identifications of the connection types, regulatory or sequestration, for varying noise levels and for the standard and optimized selection of communicating species.** Experimental perturbations of modules were simulated by decreasing the corresponding total abundances by 50%. Panels a-d refer to different noise levels of the simulated data. Each panel is organized in subpanels, which correspond to the connection coefficients in the same positions as they have in the connection matrix (e.g, in panel a in the 2^nd^ row, the 1^st^ column displays the distributions for the regulatory connection coefficient, $r_{21}$). For each connection coefficient, the bar plot shows the number of data samples where the connection was determined as regulatory connection (black color) and sequestration-induced connection (red color). On the left hand side of each subpanel, the weight parameters  $\vec{a}$ are set to zero, whereas on the right hand side, the optimal weight parameters $\vec{a}=\vec{a}^{opt}$ are chosen. The weight parameter $\vec{a}^{opt}$ was calculated separately for each data sample. Coefficients, $r_{21}$, $r_{31}$ and $r_{32}$ (black color) are the regulatory connection coefficients, and coefficients $r_{13}$, $r_{12}$ and $r_{23}$(red color) are sequestration connection coefficients (see Supplementary material Section 4).

**References**

1 Prabakaran, S., Gunawardena, J. & Sontag, E. Paradoxical results in perturbation-based signaling network reconstruction. *Biophys J* **106**, 2720-2728, doi:10.1016/j.bpj.2014.04.031 (2014).

2 Kholodenko, B. N. *et al.* Untangling the wires: a strategy to trace functional interactions in signaling and gene networks. *Proc Natl Acad Sci U S A* **99**, 12841-12846. (2002).

3 Barenblatt, G. I. *Scaling, Self-similarity, and Intermediate Asymptotics: Dimensional Analysis and Intermediate Asymptotics*. (Cambridge University Press, 1996).

4 Stelniec-Klotz, I. *et al.* Reverse engineering a hierarchical regulatory network downstream of oncogenic KRAS. *Mol Syst Biol* **8**, 601, doi:10.1038/msb.2012.32 (2012).

5 Thomaseth, C. *et al.* Impact of measurement noise, experimental design, and estimation methods on Modular Response Analysis based network reconstruction. *Scientific Reports* **8**, 16217, doi:10.1038/s41598-018-34353-3 (2018).

6 Santos, S. D., Verveer, P. J. & Bastiaens, P. I. Growth factor-induced MAPK network topology shapes Erk response determining PC-12 cell fate. *Nat Cell Biol* **9**, 324-330 (2007).

7 Santra, T., Kolch, W. & Kholodenko, B. N. Integrating Bayesian variable selection with Modular Response Analysis to infer biochemical network topology. *BMC Syst Biol* **7**, 57, doi:10.1186/1752-0509-7-57 (2013).
